# Computational load reduction of the agent guidance problem using Mixed Integer Programming

**DOI:** 10.1371/journal.pone.0233441

**Published:** 2020-06-05

**Authors:** Vinícius Antonio Battagello, Nei Yoshihiro Soma, Rubens Junqueira Magalhães Afonso

**Affiliations:** 1 Divisão de Engenharia de Computação, Instituto Tecnológico de Aeronáutica, São José dos Campos, SP, Brazil; 2 Divisão de Engenharia Eletrônica, Instituto Tecnológico de Aeronáutica, São José dos Campos, SP, Brazil; 3 Institute of Flight System Dynamics, Technical University of Munich (TUM), Garching bei München, Bavaria, Germany; University of Tehran, IRAN, ISLAMIC REPUBLIC OF

## Abstract

This paper employs a solution to the agent-guidance problem in an environment with obstacles, whose avoidance techniques have been extensively used in the last years. There is still a gap between the solution times required to obtain a trajectory and those demanded by real world applications. These usually face a tradeoff between the limited on-board processing performance and the high volume of computing operations demanded by those real-time applications. In this paper we propose a deferred decision-based technique that produces clusters used for obstacle avoidance as the agent moves in the environment, like a driver that, at night, enlightens the road ahead as her/his car moves along a highway. By considering the spatial and temporal relevance of each obstacle throughout the planning process and pruning areas that belong to the constrained domain, one may relieve the inherent computational burden of avoidance. This strategy reduces the number of operations required and increases it *on demand*, since a computationally heavier problem is tackled only if the simpler ones are not feasible. It consists in an improvement based solely on problem modeling, which, by example, may offer processing times in the same order of magnitude than the lower-bound given by the relaxed form of the problem.

## 1 Introduction

In the last decades, computers assumed an increasing proportion of tasks previously assigned to humans. Repetitive chores such as an automobile assembly in a production line can already be almost fully automated. Nevertheless, other tasks depend on human judgment, such as an aircraft guidance. In such a case, sometimes *unpredicted* events require immediate responses that demand fast replanning to obtain alternative feasible routes.

This work proposes a computationally efficient solution to the problem of calculating the trajectory of mobile agents in an environment populated by obstacles. Linear programming by itself cannot directly model this type of problem, since the solution domain is nonconvex. However, recent research on agent trajectory planning [1–10] shows that it is still possible to use linear programming subject to *integer* variables constraints to optimize the trajectory of an agent in such an environment. We use *mixed-integer linear programming* with the assumption that the totality of space available should *not* be considered as a valid possibility for an agent in a trajectory optimization problem at any and every moment. In the previous example of the car travel, the headlights privilege lighting the ground near the car over the more distant areas. Even though these also require illumination, they are not so relevant as the first ones for steering the agent.

**Fig 1:**
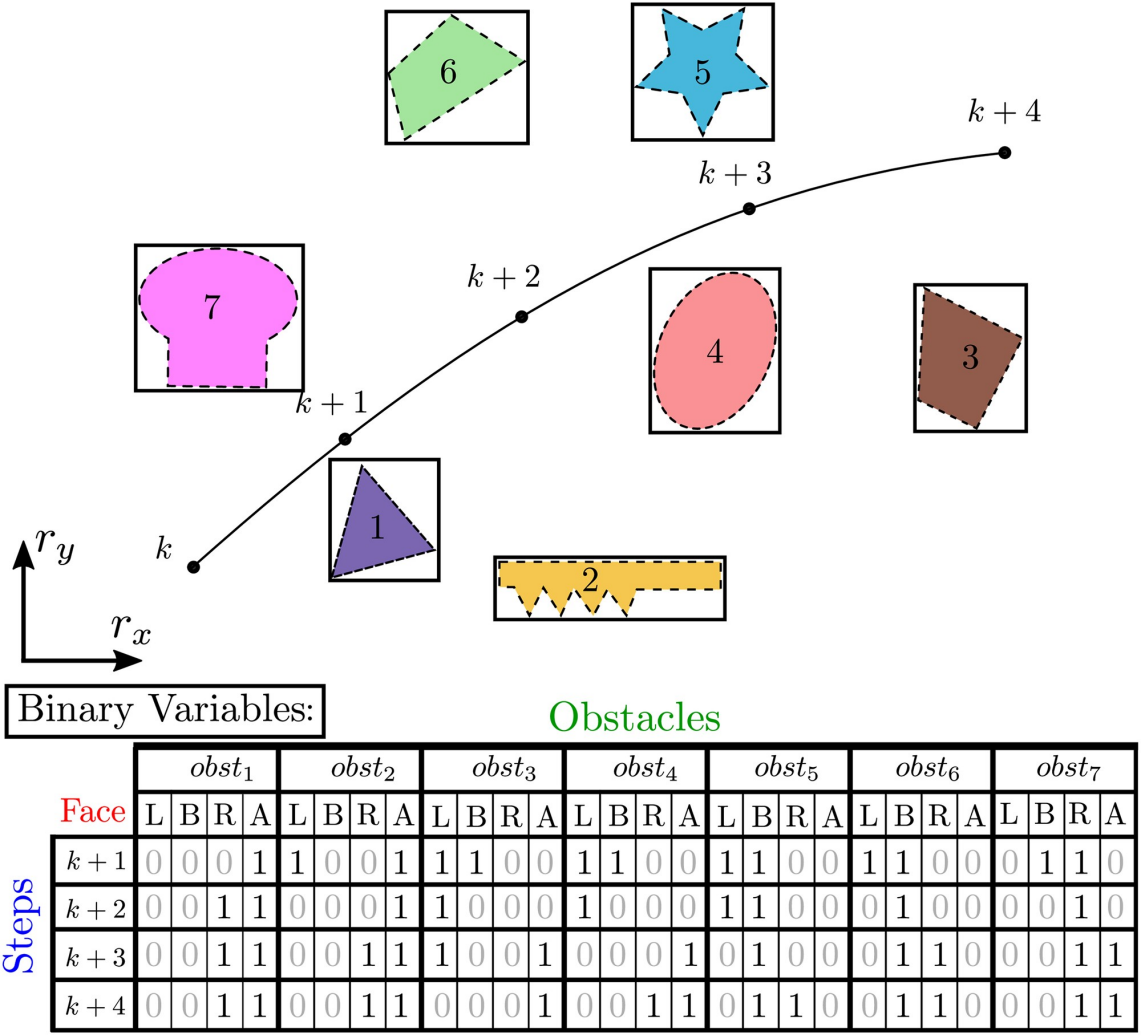
Binary variable use in a trajectory planning problem. To obtain the trajectory, the agent takes the envelope, represented in continuous lines, of each obstacle that is represented in dashed lines.

In Fig 1 we have an example of the use of binary variables in an instance of a trajectory planning problem. Initially in the position indicated by index *k*, the agent takes the envelope, represented in continuous lines, of each obstacle that is represented in dashed lines to obtain the trajectory along steps *k* + 1, *k* + 2, …, *k* + 4. In such a case, each obstacle would have a set of binary variables, indicated below the figure, that denote at each timestep whether the agent is respectively in the Left-hand side, Below, in the Right-hand side or Above each obstacle. Therefore, 28 binary variables would be necessary at every timestep to avoid the obstacle set in such a case.

This problem of obstacle avoidance is NP-hard [11–13], and the current approaches found in the scientific literature are tailored for each specific circumstance. For example, [14] obtains the optimal solution after exploring the *complete* solution space, which may demand, for complex environments, an intense computational effort that might be unattainable in a small time slice. With the assumption that it is possible to know in advance the position of fixed obstacles in the environment, we propose a strategy to obtain a sub-optimal trajectory for the agent through obstacle clustering. Basically, to the traditional mixed-integer algorithms of *obstacle* avoidance we combine an *iterative deepening* implicit tree search in the subproblems of *cluster* avoidance, with a non-increasing clustering distance. As the number of obstacles gives an upper-bound to the number of clusters, by reducing the clustering distance, we detect the best existent solution for the trajectory planning problem.

The aforementioned complexity of such problem means that, as the number of obstacle increases, trajectory optimization becomes considerably more costly. A natural plan, then, would be to reduce the number of obstacles considered throughout the process. In possession of scheme in Fig 1, it would be possible to cluster obstacles 1 and 2, once the agent moves Above both, which sets the fourth column for them along the entire trajectory. Note that this decreases the size of the problem to be solved, since a single cluster would replace both obstacles.

The immediate reaction of a human being in traffic, when driving a car and trying to escape a collision between two other cars in front of his/her own, is to promptly cluster the obstacles ahead, like other cars or lamp posts, and seek complementary territory, free of obstacles, for navigation. As previously noted, this will be one of the approaches taken in this paper. In such a case, the fastest way to compute a safe path is to cluster nearby obstacles and initially seek to drive the car into an open nearby position. The question that would remain, in such a case, would be about how far apart two obstacles must be to be clustered together, and the strategy one would choose would again follow the intuition that one should first look for safe positions in the wider open areas.

**Fig 2:**
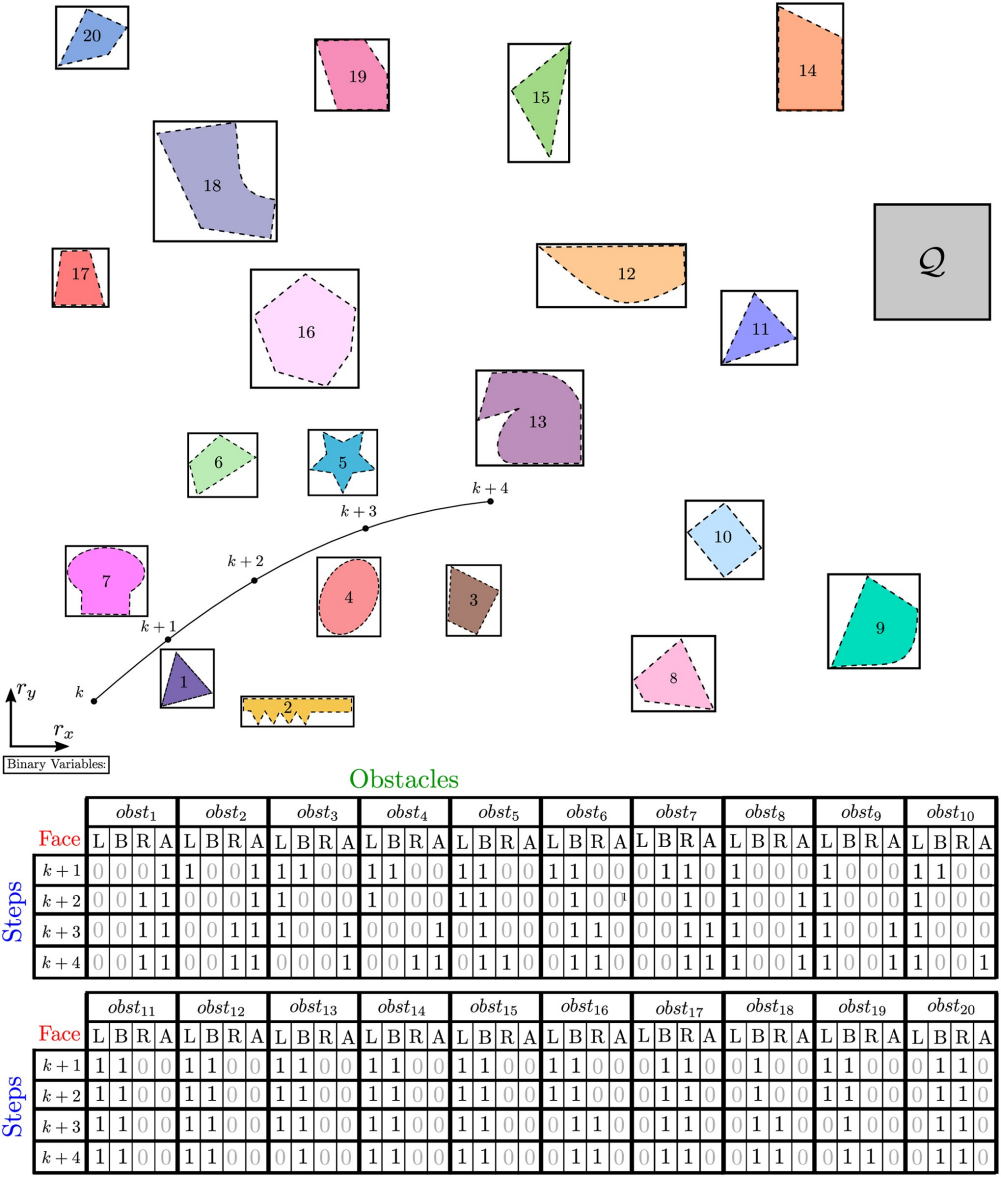
Complete map for the trajectory planning problem. The agent must attain the target set Q while avoiding obstacles.

However, other approaches can be additionally adopted to reduce the complexity of such problem. Consider in Fig 2 the complete map of the environment for the obstacles of Fig 1, where Q represents the terminal set. Among other simplifications, after step *k* + 2, the entire trajectory of the agent shall only move away from obstacles 1 and 2, for example. Considering them as obstacles to be avoided, when it is already predicted that a possible collision will not happen, is a source of computational waste. In addition, take for example obstacles 14 and 20. Right after step *k* + 1, the entire trajectory of the agent is predicted to stay below both obstacles and to the right-side of obstacle 20. The expense of limited computational resources on irrelevant calculations such as these is also useless, since they are already predicted not to aid in the computation of the final trajectory. These are a preview of the techniques we will use here.

### 1.1 Motion planning and Mixed-Integer programming

In the literature, examples of motion planning are abundant, and regardless of the medium or vehicle used, they may be split into two separate layers: *path* planning and *trajectory* planning.

*Path* planning methods build routes *without* time parametrization, as in [15] or [16], which define crossover and mutation operators on genetic algorithms, or in [17], which uses a wavelet-based decomposition for easing the computational load of a multiresolution path planner.

*Trajectory* planning methods, on the other hand, search for routes that respect the movement constraints *along time*. For example, while in [18] a linear regression model predicts the evolution of human mobility in regions of a megalopolis, [19] uses statistical modeling to investigate the hierarchical structure of accident causes in autonomous vehicles and [20] proposes a proportional-integral-derivative (PID) controller for the real-time robotic stabilization of a robotic arm to act upon a dynamically moving human with a tumor.

However, it is possible to combine both *path* and *trajectory* planning by using Mixed-Integer Programming. The MIP approach is a general problem-solving framework that involves both discrete and continuous variables. In the case of agent movement, for example, we may use the former to model the activation of brake mechanisms while the latter calculates the speed and yaw angle in a curve. By constraining the domain of some variables to integers only, it becomes an approach much more general than a Linear Program (LP).

In particular, as in [21], for certain measures only Boolean variables are considered (thus assuming values 0 or 1). This procedure allows the system dynamics to be modeled by transforming propositional logic equations that derive from it into mixed-integer inequalities, which can be computed by the existent mixed programming solvers, such as CPLEX [22], Gurobi [23], Xpress [24], Xpress-MP [25] or ParaXpress [26].

Applications of MIP in the literature are plentiful, whether in terrestrial [1, 5–8, 18, 19], aquatic [27–29], aerial [2, 4, 9, 16, 30–32] or even spatial [33] environments. For instance, the authors propose in [14] an approximate model of aircraft dynamics using *linear* constraints and they apply a MIP approach to the trajectory planning of airplanes. The model ensures collision avoidance for each aircraft and guarantees the desired hard constraints fulfillment. Then, [34] applies a constraint tightening strategy to obtain a robust solution that guarantees finite-time arrival into an arbitrary target set. In spite of unknown disturbances, the central idea is to hold a “border” for feedback action as time goes by.

We assume in this manuscript that the computation time demanded in trajectory planning grows with the number of binary variables used for obstacle avoidance. This is not always valid, but can be used as a rule-of-thumb for improving computational performance. In [35], such number is used to express the complement of the polytope regions, which associate a unique number to each obstacle. As the sequence of the number powers in base 2 is super increasing, according to [36], any integer can be coded in log_2_(*N* + 1) binary digits, which is the number of binary variables necessary to distinguish between *N* different regions.

In other words, [35] sets a *global limit* to the encoding itself. It is important to recall that the *direct* MIP solution for the trajectory optimization problem in an environment with obstacles is hard because in every timestep each obstacle face introduces non-convexity into the solution space [11–13]. As a consequence, for every obstacle with *N*_*f*_ faces, *N*_*f*_ non-convex constraints shall be added at each timestep to ensure that the desired trajectory remains outside the obstacle. As a result, to obtain further processing speedup, every technique obtained after [35] should involve a *pre*-processing step, performed separately with respect to the optimization.

For example, [37] proposes a strategy which requires a pre-planned path to define intermediate target sets, known as *waypoints*. Alternatively, [38] obtains large ellipsoidal regions of convex obstacle-free space in intricate environments with a greedy convex segmentation technique and [31] provides an entirely collision-free path with reduced number of integer variables. In turn, [39] divides large and complex environments into smaller segments through many pre-processing steps, [40] uses convex optimization to obtain target defect areas and [41] builds a convex lifting which partitions the space and descends to convex optimization.

However, we often demand a *dynamically*-built method in actively-changing scenarios, and this is a special contribution offered here. For instance, [42] develops a resembling approach with a three-stage algorithm: first it computes a collision-free path through the environment, next it generates convex polytopes that contain such route and then it poses a MIP to determine the dynamically feasible path. Yet, we know that peripheral, possibly distant, obstacles are not as significant as those circumvented along a path, which opens way to achieve performance gains in scenarios with many obstacles.

Additionally, it is worth noting that the approaches here presented do *not* count on *pre*-processed trajectory segments. But, as it will be clear, the results we achieved introduce a feasible and versatile way of solving the trajectory planning problem, more specifically through obstacle clustering.

## 2 Materials and methods

In this article we study the problem of maneuvering an agent into a target region in a two-dimensional environment. The agent has state *x*, compounded by its position *r* and velocity *v*, and must not collide against obstacles. The dynamics of the agent is represented in Eq (1):

$$\begin{array}{c} x[k+1]=Ax[k]+Bu[k] \end{array}$$ (1)

Model Predictive Control (MPC) is used to perform the maneuver of the system in Eq (1). The core of the control problem is to choose optimally the predictions $\hat{x}\left[k+j|k\right]$ and the corresponding $\hat{u}\left[k+j|k\right]$ for each timestep *j* ∈ {0, …, *N*}. To attain the target set Q in finite time, we take the horizon length *N*[*k*] as a decision variable within the optimization, which represents the predicted time of entering it.

This paper deals with the task of maneuvering an agent with linear dynamics minimizing the cost function

$$\begin{array}{c} J[x[k]]=\min_{\hat{u},\hat{x},N\left[k\right]}\sum_{j=0}^{N[k]}(1+\gamma ∥{\hat{u}[k+j|k]∥}_{1}) \end{array}$$ (2)

subject to

$$\hat{x}[k+j|k]\in X,$$ (3a)

$$\hat{u}[k+j|k]\in U,$$ (3b)

$$\hat{r}[k+j|k]\notin O,$$ (3c)

$$\hat{x}[k+N[k]+1|k]\in Q[N[k]+1],$$ (3d)

$$\hat{x}[k+j+1|k]=A\hat{x}[k+j|k]+B\hat{u}[k+j|k],$$ (3e)

j=0,1,…,N[k]. (3f)

**Fig 3:**
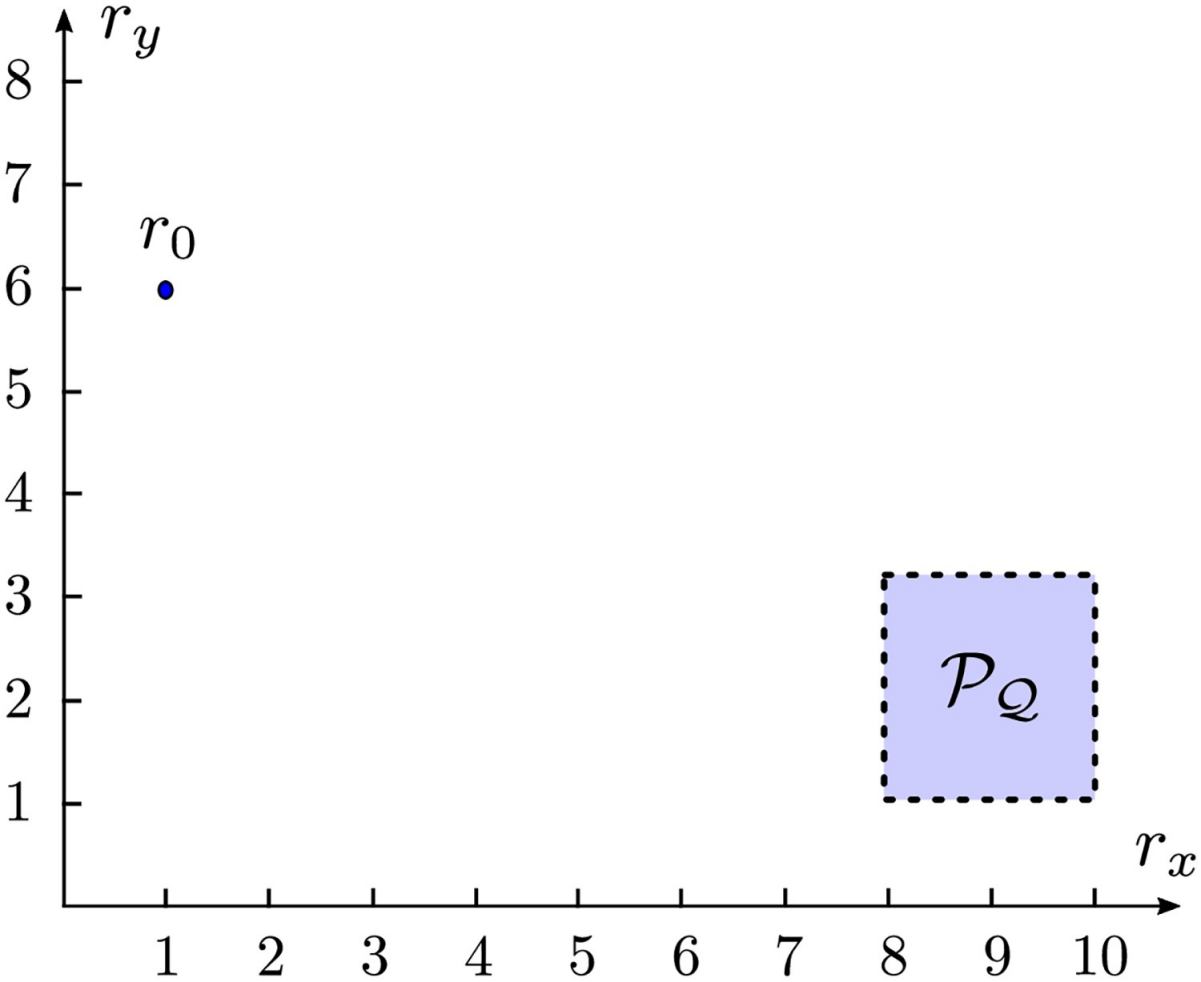
Example of a trajectory planning environment. The agent is initially in *r*_0_ in a trajectory planning problem, and in this case, *x*_0_ = [1 0 6 0]^*T*^ and Q could be defined by 8 *m* ≤ *r*_*x*_ ≤ 10 *m*, 1 *m* ≤ *r*_*y*_ ≤ 3 *m*, |*v*_*x*_|≤0.01 *m*/*s* and |*v*_*y*_|≤0.01 *m*/*s*, for example.

In Fig 3 we have a representation of the projection $P_{Q}$ of Q onto the position space.

### 2.1 Time minimization

It is possible to transform a *variable* horizon problem involving time minimization into a *fixed* horizon one with the use of integer variables. With the assumption of a polytopic set Q of terminal constraints,

$$\begin{array}{c} Q=\{x|D^{m}x\leq H^{m}\} \end{array}$$ (4)

in which $D^{m}\in R^{N_{Q}\times 4}$ and $H^{m}\in R^{N_{Q}}$.

To perform the time minimization task, the terminal constraint in Eq (3d) can be rewritten by making use of auxiliary binary variables *b*[*j*] which are determined as *b*[*j*] = 1 if *N*[*k*] = *j* and *b*[*j*] = 0 otherwise. The following constraints impose, for some *j* such that *b*[*j*] = 1 and in interplay with the cost function to be redefined in Eq 7, that the agent must be within Q, where *N*_*s*_ is a fixed maximum horizon that must be larger than than the optimal *N*[*k*]:

$$\begin{array}{c} d_{i}^{m}\hat{x}\left[k+j+1|k\right]\leq h_{i}^{m}+M_{t}\left(1-\hat{b}\left[k+j+1|k\right]\right),\;0\leq j\leq N_{s}-1 \end{array}$$ (5)

with $d_{i}^{m}$ and $h_{i}^{m}$ corresponding respectively to *i*-th line of matrix *D*^*m*^ and to the *i*-th component of vector *H*^*m*^.

To ensure that the binary variable *b* assumes the unitary value only once over the horizon *N*_*s*_, the following constraint is defined:

$$\begin{array}{c} \sum_{j=0}^{N_{s}-1}\hat{b}\left[k+j+1|k\right]=1 \end{array}$$ (6)

As a result, the variable-horizon cost function in Eq (2) can be rewritten by using a binary variable vector *b* to ensure the correctness in a fixed horizon approach:

$$\begin{array}{c} J(x[k])=\min_{\hat{u},\hat{x},\hat{b}}\sum_{j=0}^{N_{s}-1}[\left(j+1\right)\hat{b}\left[k+j+1|k\right]+\gamma ∥{\hat{u}[k+j|k]∥}_{1}] \end{array}$$ (7)

where $\hat{u}\left[k+j|k\right]=0$ for *j* > *N*[*k*], *i.e*. the control is zero for all steps beyond the chosen maximal horizon $\bar{N}$. This is a consequence of the relaxation of Eqs (3a) and (3c) after *N*[*k*] in the minimization of Eq (2).

**Fig 4:**
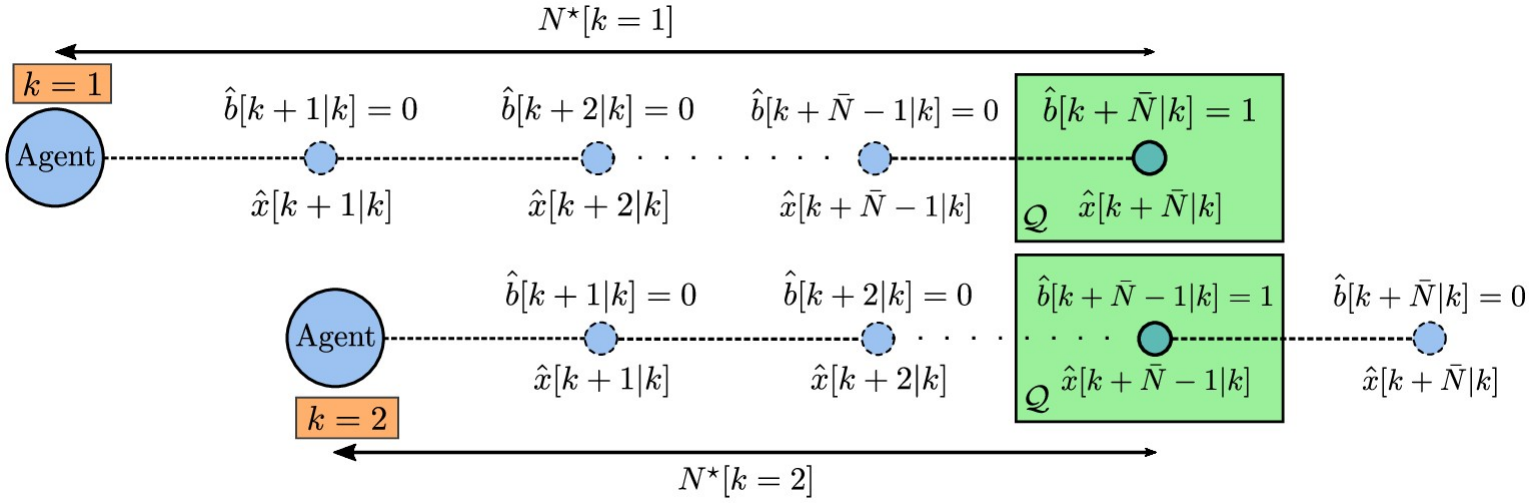
Constraint relaxation scheme in a variable horizon problem. For *k* = 1, the binary variable $\hat{b}\left[k+\bar{N}|k\right]$ is set in the horizon *N*^⋆^[*k* = 1], when $\hat{x}\left[k+\bar{N}|k\right]\in Q$. In *k* = 2, the agent repeats another instance of the same variable horizon problem, which now sets variable $\hat{b}\left[k+\bar{N}-1|k\right]$, as $\hat{x}\left[k+\bar{N}-1|k\right]\in Q$ for *N*^⋆^[*k* = 2].

In Fig 4 there is a scheme that represents the relaxation of the time minimization binary variables in a trajectory planning problem. Note that *N*^⋆^[*k* = 2] = *N*^⋆^[*k* = 1] − 1, *i.e*. the optimal horizon at *k* = 2 is one unit smaller than the one at *k* = 1. The constraints in Eq (5) are relaxed for *j* > *N*^⋆^[*k* = 1], once $\hat{b}\left[k+\bar{N}|k\right]=1$, and for *j* > *N*^⋆^[*k* = 2], once $\hat{b}\left[k+\bar{N}-1|k\right]=1$. To accomplish it, the scalar *M*_*t*_ must be chosen such that $M_{t}>d_{i}^{m}\hat{x}-h_{i}^{m}$, for all admissible *x* [21]. That is, *M*_*t*_ must be chosen large enough for every *x* reachable in $\bar{N}$ steps, to serve as a barrier that allows numerical solvers to correctly modulate the domains of action of the continuous variables through the binary variables $\hat{b}\left[k+j|k\right],0\leq j\leq N_{s}-1$. Such variables act as sidings that separate the action domain of each continuous instance of the problem and allow the solver to perform *global* optimization calculations by evaluating multiple local domains at once. Note also that, for *k* = 2, the binary variable $\hat{b}\left[k+\bar{N}|k\right]$, which marks the arrival to Q in the last step of the simulation horizon, is clear, so that Eq (7) ≡ Eq (2) in this problem instance.

### 2.2 Obstacle avoidance

The optimization of trajectories in two-dimensional territories, clear of obstacles, presents an inherently *convex* search space, but the insertion of an obstacle into the region may render the problem not convex. Hence, we describe the commonly adopted remodeling that follows.

As a common requirement in an agent guidance problem, its formulation includes an obstacle avoidance task. Any polytopic obstacle O can be represented by:

$$\begin{array}{c} O=\{x|S^{O}x\leq C^{O}\} \end{array}$$ (8)

with $S^{O}\in R^{N_{f}N_{s}\times 4}$ and $C^{O}\in R^{N_{f}N_{s}}$.

We avoid collisions against obstacles by imposing that, at each time step, the position of the agent is outside of at least one face of each obstacle. This is done through the binary variable $b_{f,j}^{O}$, which sets if the agent is outside face *f* of obstacle O at time step *k* + *j*.

As we need $x\left[k\right]\notin O\subset R^{4},\forall k\in N$, we should have

$$s_{f}^{\text{O}}\hat{x}[k+j|k]\geq c_{f}^{\text{O}}+(b_{f,j}^{\text{O}}-1)M_{o},1\leq j\leq N_{s}$$ (9a)

$$\sum_{f=1}^{N_{f}}b_{f,j}^{\text{O}}\geq 1$$ (9b)

where $s_{f}^{O}$ and $c_{f}^{O}$ correspond both to the *f*-th line of matrix S^O^ and to the *f*-th element of vector *C*^O^, respectively.

Obstacle avoidance in a trajectory planning problem can be attained by using the strategy of Alg 1. Initially we load the simulation parameters, and while the agent state *x*[*k*] does not attain Q, based on *N*_*s*_ and the positions of the obstacle set *R*_*ob*_, we assemble the matrices **A** and **B** that contain the constraints of the problem, plan the trajectory by solving a traditional MIP problem, and finally evaluate and update the system state.

**Alg 1. Closed-loop receding horizon maneuvering with Obstacle Avoidance**.

**Input**: $x\left[k\right],R_{ob},N_{s},Q$

**Output**: *x*[*k* + 1]

1: *Load Simulation Parameters*

2: **while** x[k]∉Q **do**

3:  [**A**, **B**]←*Assemble Problem Matrices*(*R*_*ob*_, *N*_*s*_)

4:  *u*[*k*]← *Solve MIP Problem*(**A**, **B**)

5:  *x*[*k* + 1]←**A** *x*[*k*]+ **B** *u*[*k*]

6:  *k* ← *k* + 1

7: **end while**

**Fig 5:**
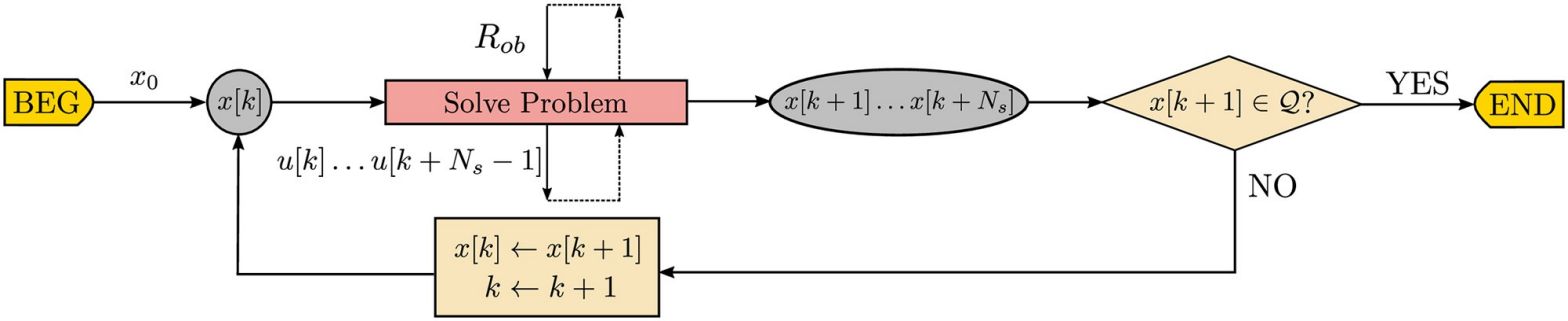
Data flowchart of the closed-loop receding horizon maneuvering. The agent solves the optimization problem to avoid the obstacle set *R*_*ob*_ and applies the first element *u*[*k*] of the control sequence *u*[*k* + *i*], 0 ≤ *i* ≤ *N*_*s*_ − 1.

In Fig 5 there is a flowchart of the main variables produced throughout the different steps of the traditional obstacle avoidance algorithm. The regular data flow is represented by the loop over state *x*[*k*] until the next state x[k+1]⊆Q.

However, as each obstacle partitions the search space into *N*_*f*_ disjoint regions, the overall performance in environments with tens of obstacles is severely deteriorated with regard to the initial obstacle-free case.

### 2.3 Inter-sample avoidance

In addition to guaranteeing obstacle avoidance in the sampled time steps, inter-sample avoidance is achieved by applying the avoidance constraints from step *j* + 1 at the preceding timestep *j*, as proposed by [43]. This imposes additional constraints to the problem, but employs no additional binary variables, as Eq (10) shows.

$$\begin{array}{c} s_{f}^{O}\hat{x}\left[k+j|k\right]\;\geq \;c_{f}^{O}+\left(b_{f,j+1}^{O}-1\right)M_{o},0\leq j\leq N_{s}-1 \end{array}$$ (10)

### 2.4 Considering fuel expense

In order to cope with the one-norm of the control variables in Eq (7), which penalizes fuel expense, we add a set of auxiliary variables *ϵ* which are constrained [44]. As a result, in the optimization we obtain a compromise solution between *fuel expense* and *time minimization*. Then, we add the following constraints to the problem:

$$-{\hat{\epsilon }}_{x}[k+j|k]\leq {\hat{u}}_{x}[k+j|k],$$ (11a)

$$-{\hat{\epsilon }}_{y}[k+j|k]\leq {\hat{u}}_{y}[k+j|k],$$ (11b)

$$-{\hat{\epsilon }}_{x}[k+j|k]\leq -{\hat{u}}_{x}[k+j|k],$$ (11c)

$$-{\hat{\epsilon }}_{y}[k+j|k]\leq -{\hat{u}}_{y}[k+j|k],$$ (11d)

$$0\leq j\leq N_{s}-1$$

The previous cost function given by Eq (7), then, reaches its final form in Eq (12)

$$\begin{array}{c} J(x[k])=\min_{\hat{\epsilon },\hat{x},\hat{b}}\sum_{j=0}^{N_{s}-1}[\left(j+1\right)\hat{b}\left[k+j+1|k\right]+\gamma ({\hat{\epsilon }}_{x}\left[k+j|k\right]+{\hat{\epsilon }}_{y}\left[k+j|k\right])], \end{array}$$ (12)

subject to the constraints in Eqs (3a), (3b), (5), (6), (9), (10) and (11), for 0 ≤ *j* ≤ *N*_*s*_ − 1.

## 3 Obstacle clustering algorithm

### 3.1 Graph theory background

In this work, we assume that each obstacle is a static object with known coordinates. Let $R_{ob}\subset R^{N_{f}N_{ob}}$ be the the smallest and largest coordinates of the projection of each obstacle of O into the position space. Let also T be a territory with target set Q in an environment around the agent that contains *N*_*ob*_ obstacles in a given clustering region, with a corresponding maximum clustering distance $d_{c}^{max}$, chosen by the trajectory planner.

Then, T can be mapped into an undirected graph G=(O,Ω), where the order of the graph is $|O|=N_{ob}$ and |Ω| indicates the number of obstacle pairs that maintain a distance $d\leq d_{c}^{max}$. As an example, two nodes $O_{1}$ and $O_{2}$ corresponding to obstacles *O*_1_ and *O*_2_ in O with points *p*_1_ ∈ *O*_1_ and *p*_2_ ∈ *O*_2_ will be connected if

$$\begin{array}{c} d=\min_{p_{1}\in O_{1},p_{2}\in O_{2}}(∥{r_{d}\left(p_{1}\right)-r_{d}(p_{2})∥}_{2})\leq d_{c}^{max} \end{array}$$ (13)

where the function *r*_*d*_(⋅) returns the position of ⋅.

The fulfilling of Eq (13) for each pair of obstacles is recorded by the adjacency matrix A, a square matrix formed by *N*_*ob*_ lines in which *a*_*ij*_ = 1 if and only if the vertices (obstacles, in this case) $O_{i}$ and $O_{j}$ are connected, and *a*_*ij*_ = 0, otherwise.

**Definition 1**. *Let* X=(A∨I) *for the identity matrix I, where ∨ is the element-wise OR operation. Then, if we take* $X^{‡}$ *for* ‡∈N, *where* $X^{‡}$ *is the* ‡-*th power of matrix* $X={\left(x\right)}_{ij}$, *each element* ${\left(x^{r}\right)}_{ij}=1$ *if and only if there is a path between the obstacles corresponding to nodes* $O_{i}$ *and* $O_{j}$ *with length l* ≤ *r* [45].

**Definition 2**. *Let* $C={\left(c\right)}_{ij}$, *in which* $c_{ij}={\left(x^{N_{d}-1}\right)}_{ij}$, *where N*_*d*_ *is the number of nodes in the graph. Then c*_*ij*_ = 1, ∀*i*, *j* ≤ *n if and only if there is a path from* $O_{i}$ *to* $O_{j}$ *in graph* G. C *is known as the connectivity matrix of* G.

If lines—or columns, as A and C are symmetrical for undirected graphs—*i* and *j* in C are equal, then $O_{i}$ and $O_{j}$ will have a path to the same nodes. In this case, they will belong to the same connected component, or *cluster*, as we will name it from now on.

**Remark 1**. *To verify if the obstacles corresponding to nodes* $O_{i}$ *and* $O_{j}$ *belong to the same cluster, it is enough to compare the i-th and j-th lines (or columns) in C. If they are equal, then the obstacles belong to the same cluster*.

**Fig 6:**
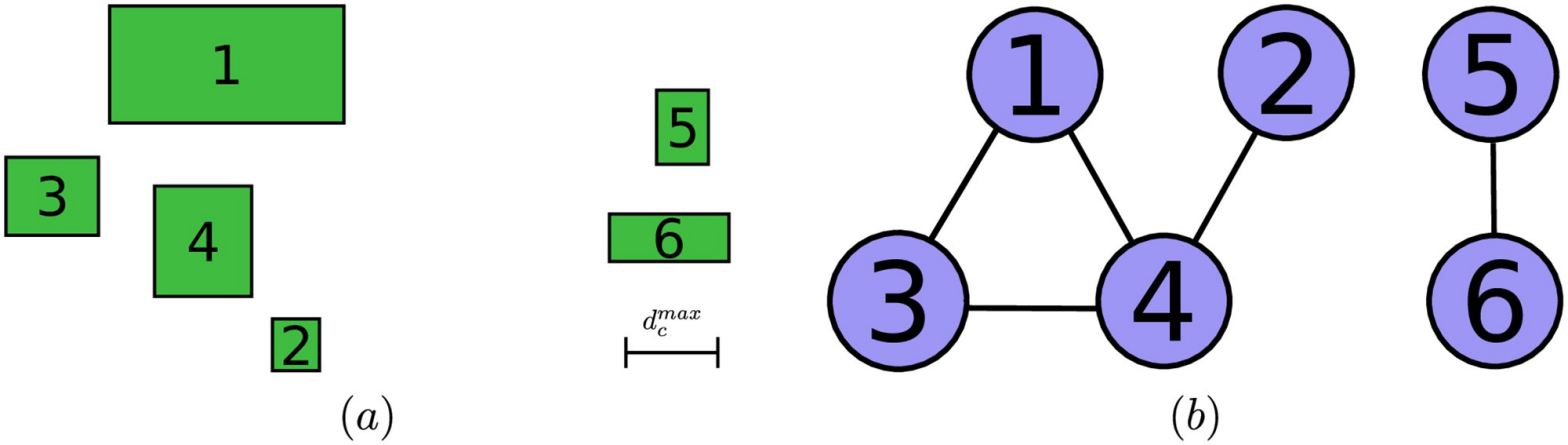
Example of the graph extracting from a spatially located set of obstacles. (a): Spatial obstacle distribution. (b): Graph of the obstacle interconnection.

As an example, the terrain that contains a set of *N*_*ob*_ = 6 *obstacles* (Fig 6a), can be represented by the graph in Fig 6b, which is described both by the adjacency matrix A and the connectivity matrix C in Eq (14).

$$\begin{array}{c} A=[\begin{array}{cccccc} 0 & 0 & 1 & 1 & 0 & 0\\ 0 & 0 & 0 & 1 & 0 & 0\\ 1 & 0 & 0 & 1 & 0 & 0\\ 1 & 1 & 1 & 0 & 0 & 0\\ 0 & 0 & 0 & 0 & 0 & 1\\ 0 & 0 & 0 & 0 & 1 & 0 \end{array}] \end{array},\begin{array}{c} C=[\begin{array}{cccccc} 1 & 1 & 1 & 1 & 0 & 0\\ 1 & 1 & 1 & 1 & 0 & 0\\ 1 & 1 & 1 & 1 & 0 & 0\\ 1 & 1 & 1 & 1 & 0 & 0\\ 0 & 0 & 0 & 0 & 1 & 1\\ 0 & 0 & 0 & 0 & 1 & 1 \end{array}] \end{array}.$$ (14)

The resulting clustering procedure is computationally efficient, since in order to obtain the connected components of O it is enough to assemble A, with a simple set of comparisons between the obstacle positions, and to obtain C, with only the use of matrix multiplications.

**Alg 2. Copertinence test of two obstacles in the same cluster**.

**Input**: C,i,j

**Output**: *SameCluster*

1: **if** C(i,:)=C(j,:) **then**

2:  *SameCluster* ← True

3: **else**

4:  *SameCluster* ← False

5: **end if**

From Eq (13), the interobstacle distance is obtained as the Hausdorff distance between the sets defined by the points inside obstacles *O*_*i*_ and *O*_*j*_.

Here, as *R*_*ob*_ contains the stacked coordinates in the *x*- and *y*-axis of the lower left-hand side and the upper right-hand side corners of every non-intersecting obstacle, to determine the interobstacle distances *Δr*_*o*_ between every two obstacles, it is enough to make a simple set of comparisons and operations with the position of the corners of every obstacle pair. It is worth noting that, as long as the environment does not change and is supposed to be static, *Δr*_*o*_ can be computed *offline* and used throughout the agent movement, once it depends only on the environment topology. It is also worth remarking that the clustering distance *d*_*c*_ is the generalization of $d_{c}^{max}$ for multiple obstacles, as next section will highlight.

It is possible to check the copertinence of two obstacles to the same cluster with Alg 2, and there is a representation of this clustering strategy in Alg 3. Here, *Δr*_*ao*_ is an array in which each element *Δr*_*ao*_[*i*] is the value of the distance between the agent and obstacle *O*_*i*_. The main idea is to obtain the matrix C, matrix compounded by *N*_*c*_ lines and *N*_*ob*_ columns which indicates for the agent position *r* and the obstacle set position *R*_*ob*_ if two obstacles are neighbors in a given clustering region. The first *for* loop identifies the innermost clustering region each obstacle belongs to in order to set the respective clustering distances *d*_*c*_[*μ*], for each *μ*-th obstacle. The next two nested *for* loops build the adjacency matrix A and as there are no loops in G, we must add A to the identity matrix before raising it to (*N*_*ob*_ − 1) − *th* power, so as to propagate the links that pass through a node and obtain the maximum number of obstacles clustered together in the environment. Next we obtain the connectivity matrix C and, as we are only interested in the identification of the connected components of obstacles, we finally set the connectivity of these as 1.

**Alg 3. Obstacle clustering**.

**Input**: *r*, *R*_*ob*_, *R*_*ic*_, *R*_*sc*_, *d*_*ic*_, *d*_*sc*_, *d*_*oc*_, *N*_*ob*_

**Output**: C

1: *Δr*_*ao*_← get agent-obstacle distances (*r*, *R*_*ob*_)

2: **for all** *i* ≤ *N*_*ob*_ **do**

3:  **if** *Δr*_*ao*_[*i*]<*R*_*ic*_ **then**

4:   *d*_*c*_[*i*]←*d*_*ic*_

5:  **else if** *Δr*_*ao*_[*i*]<*R*_*sc*_ **then**

6:   *d*_*c*_[*i*]←*d*_*sc*_

7:  **else**

8:   *d*_*c*_[*i*]←*d*_*oc*_

9:  **end if**

10: **end for**

11: *Δr*_*o*_← get interobstacle distances (*R*_*ob*_)

12: **for all** *i* ≤ *N*_*ob*_ **do**

13:  **for all** *j* ≤ *N*_*ob*_ **do**

14:   **if** *Δr*_*o*_[*i*, *j*]<min(*d*_*c*_[*i*], *d*_*c*_[*j*]) **then**

15:    A[i,j]←1;

16:    A[j,i]←1;

17:   **else**

18:    A[i,j]←0;

19:    A[j,i]←0;

20:   **end if**

21:  **end for**

22: **end for**

23: $C\leftarrow {\left(A+I\right)}^{N_{ob}-1}$

24: **for all** *i* ≤ *N*_*ob*_ **do**

25:  **for all** *j* ≤ *N*_*ob*_ **do**

26:   **if** C[i,j]>1 **then**

27:    C[i,j]←1

28:   **end if**

29:  **end for**

30: **end for**

These tools allow us to build a clustering strategy that directly associates the reduction in the computational effort to the trajectory planning problem in the presence of obstacles, through a more refined clustering only for closer obstacles. This is the subject of the next subsection.

### 3.2 Dynamic obstacle clustering strategy

The approaches proposed by [30, 38] consider the interobstacle relative positions to find convex regions of obstacle free space, which is analogous to considering the *interobstacle distances* *Δr*_*o*_ to perform the clustering procedure, before optimization *per se*. In the trajectory planning problem, the computational speedup that these approaches obtain is a consequence of taking into account, in the avoidance modeling, the clusters themselves instead of the obstacles. As those potentially consist in a smaller number regarding these, less binary avoidance variables and constraints will possibly be used, fact which produces a problem that can be solved with less computational effort.

This work adds to this rationale the relative agent-obstacle position *Δr*_*ao*_. The strategy we adopt segments this distance in regions around the agent, and flexibilizes the effective interobstacle clustering distance *d*_*c*_ in each of these regions. For obstacles in a region closer to the agent, a more subtle clustering with a smaller value of *d*_*c*_ is adopted. For more distant obstacles, a coarser clustering may be used with a greater value of *d*_*c*_, as these obstacles are not in the immediate future of the agent. In this way, two obstacles can be clustered together while distant from the agent, but they would possibly separate into distinct clusters if the agent approaches them along its trajectory. Once less binary avoidance variables and constraints would be used compared to the single clustering distance case, a smaller total computational effort would be necessary, saving computational time to find the desired trajectory.

To make the exposition easier, here we will consider only polytopic (more specifically, rectangular) obstacles with sides parallel to the axes. Nonetheless, it must be remarked that the technique proposed in the present paper can be extended for any form of polygonal obstacles.

#### 3.2.1 Close obstacles clustering

**Fig 7:**
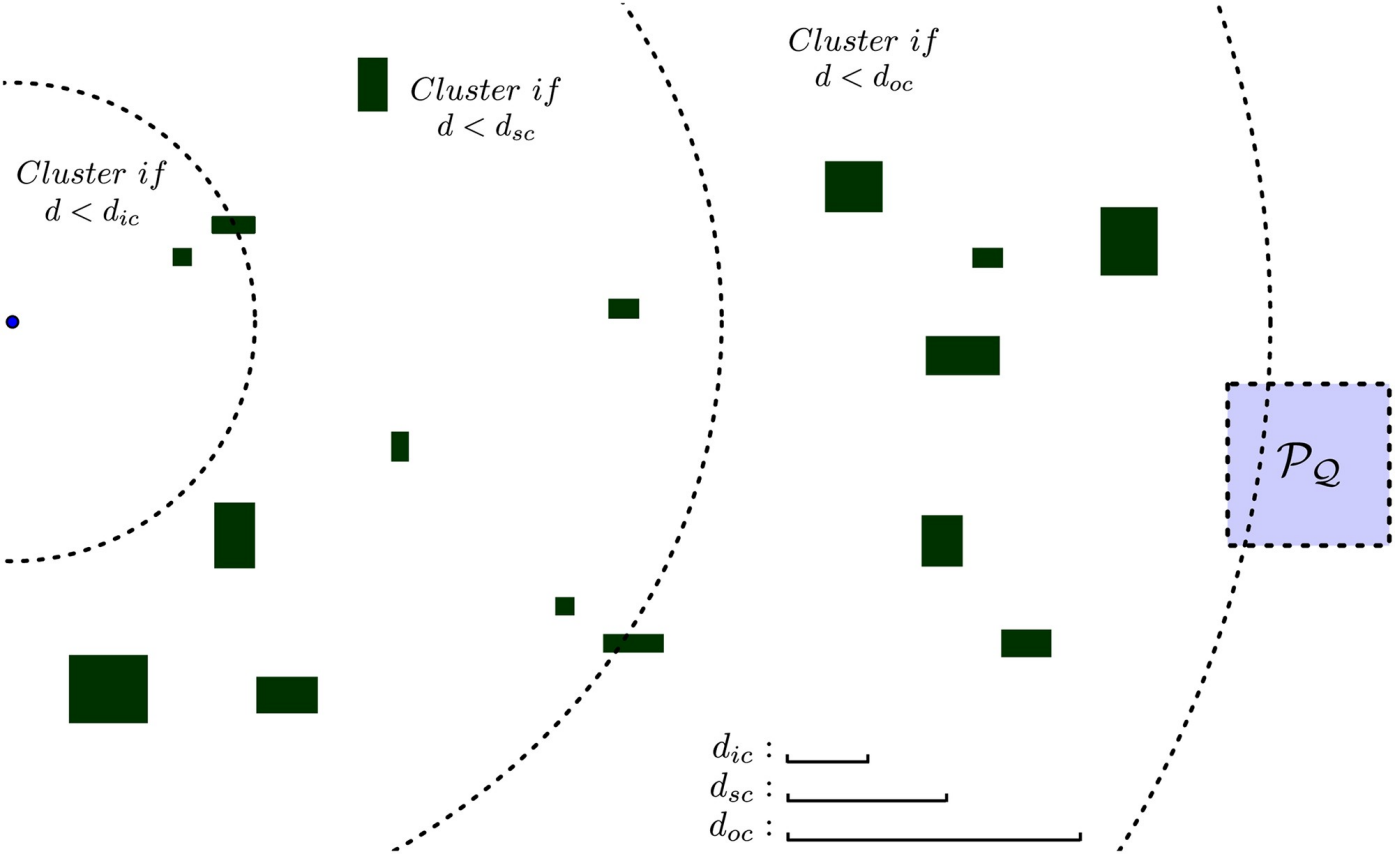
Typical scenario in a trajectory planning problem. The agent is depicted as a *blue* dot, the obstacles in *green* and the target set *R*_*f*_ as a *blue* square.

This section describes the first clustering strategy this paper proposes. A typical scenario for trajectory planning is shown in Fig 7. Each clustering region is represented as a circle around the agent with the respective clustering radii *R*_*ic*_, *R*_*sc*_ and *R*_*oc*_. The clustering distances *d*_*ic*_, *d*_*sc*_ and *d*_*oc*_ are depicted at the bottom of the figure.

After obtaining the connectivity matrix C, which describes the interconnections among close obstacles, the next step is to identify the coordinates of the clusters, as Alg 4 shows. This is done by *boxing the convex hull* of the connected components of obstacles in the environment, according to the value of *d*_*c*_ chosen in the clustering algorithm. After that, it is just a matter of solving the optimization problem by considering *cluster* avoidance, with coordinates *R*_*κ*_, instead of *obstacle* avoidance, with coordinates *R*_*ob*_, with the constraints proposed in the previous section.

**Alg 4. Cluster coordinates extraction**.

**Input**: $R_{ob},C,N_{c},N_{ob}$

**Output**: *R*_*κ*_

1: *c* ← 1

2: *o* ← 1

3: $\tilde{C}\leftarrow \;Extract\;Connected\;Components\left(C\right)$

4: **while** *c* ≤ *N*_*c*_ **do**

5:  **while** *o* ≤ *N*_*ob*_ **do**

6:   **if** $\tilde{C}\left[c,o\right]>0$ **then**

7:    *CH* ← *convex hull*(*c*, *o*)

8:    *R*_*κ*_ ← *bounding box*(*CH*)

9:   **end if**

10:   *o* ← *o* + 1

11:  **end while**

12:  *c* ← *c* + 1

13: **end while**

**Fig 8:**
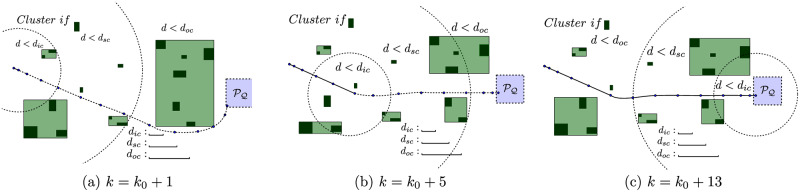
Scheme of the close obstacles clustering strategy. (a): Obstacles are grouped in 7 clusters. (b): Obstacles are grouped in 9 clusters. (c): Obstacles are grouped in 8 clusters.

The scheme in Fig 8 represents this strategy with three different agent positions. Initially, the agent estimates a path that goes down the largest cluster, to the east (Fig 8a). However, as the agent starts moving, some obstacles of this cluster enter into the *surroundings-zone* (Fig 8b) and the algorithm splits it up in two smaller clusters, which frees a corridor for the agent to reach the target (Fig 8c).

We depict the algorithm that represents this strategy in Alg 5. Basically, to the usual problem of trajectory planning, with instructions shown in black, we include an obstacle clustering phase, shown in blue. Initially, we load the simulation parameters, *i.e*. obstacle positions, clustering distances, and so on, and obtain the Adjacency and Connectivity matrices A and C. In possession of C, it is straightforward to obtain the cluster configuration *R*_*κ*_ and perform a traditional trajectory planning to reach the target region $P_{Q}$.

**Alg 5. Closed-loop receding horizon maneuvering with Close Obstacles Clustering Algorithm**.

**Input**: *x*[*k*], *R*_*ob*_, *R*_*f*_

**Output**: *x*[*k* + 1]

1: *Load Simulation Parameters*

2: **while** x[k]∉Q **do**

3:  *R*_*k*_ ← *Cluster Obstacles in Neighborhood*(*r*[*k*], *R*_*ob*_, *d*_*c*_, *R*_*ic*_, *R*_*sc*_, *R*_*oc*_)

4:  [**A**, **B**]←*Assemble Problem Matrices*(*R*_*k*_, *N*_*s*_)

5:  *u*[*k*]← *Solve MIP Problem*(**A**, **B**)

6:  *x*[*k* + 1]←**A** *x*[*k*]+ **B** *u*[*k*]

7:  *k* ← *k* + 1

8: **end while**

**Fig 9:**
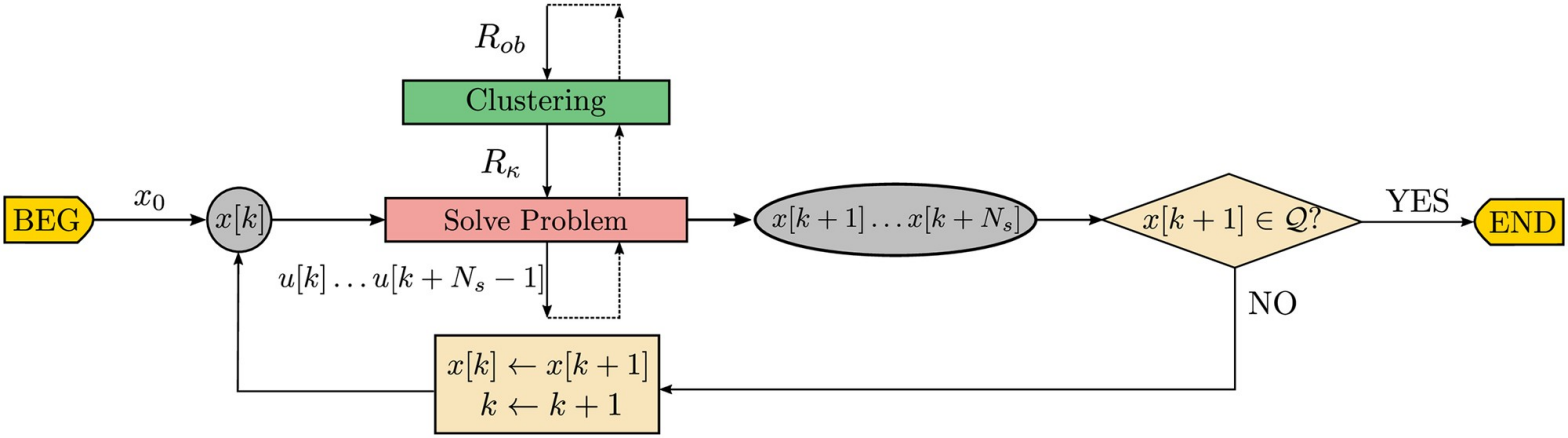
Data flowchart of the closed-loop receding horizon maneuvering with close obstacles clustering algorithm.

In Fig 9 there is a flowchart of the main variable data types produced throughout the different steps of the Close Obstacles Clustering algorithm. To the regular flow of an obstacle avoidance algorithm, represented by the feedback on state *x*[*k*], we prepend the *Clustering* step, represented by the *green* box, which produces the coordinates of the cluster configuration *R*_*κ*_ from the obstacle positions *R*_*ob*_. Notice that the complete underlying clustering logic described along this section in Alg 2, 3, 4 and 5 is here implicit, and that in Fig 9 both the iterations of the upper minor *Clustering* loop as the lower major update loop repeat until x[k+1]∈Q.

This clustering component deals only with the *current* positions of the agent and the obstacles, *i.e*. it affects the *present* situation of the system.

## 4 Complementary strategies for reducing the number of obstacles

In this section we propose additional strategies to reduce the number of obstacles along the trajectory planning problem. Specifically, both position history as forward movement predictions can help speedup the trajectory planning process.

### 4.1 Bygone obstacles rebuttal strategy

After optimization, a trajectory between the initial position *r*[*k*] and the target set is found. If all the predicted positions of the agent are closer to $P_{Q}$ than a given obstacle whose projection into the position space is given by O_*b*_, then such obstacle may be replaced by a single constraint independent of the obstacle avoidance binary variables, which may then be removed from the optimization problem from *k* + 1 onwards. In such a case, we first determine a straight-line *perpendicular* to the segment $\bar{pf}$ connecting point *p* in *O*_b_ and point $f\in P_{Q}$ that produces the minimum distance between O_*b*_ and $P_{Q}$, and then we translate this straight-line to pass at the point *p*. The half-plane defined by this line that contains the current position is guaranteed to contain all predicted positions by construction and defines the *hard* constraint that can replace the bygone obstacle in the subsequent trajectory, and ensures that the agent does not collide with it.

Therefore, we can replace the obstacle of projection O_*b*_ itself (and its binary avoidance variables $b_{f,k}^{O}$) by *hard* constraints

$$\begin{array}{c} s^{O_{b}}x\left[k+j+1|k\right]\geq c^{O_{b}},\;\;\;0\leq j\leq N_{s}-1 \end{array}$$ (15)

that ensure the agent will be confined to a region *away* from the obstacle. Here, $s^{O_{b}}$ contains the coefficients of the straight-line perpendicular to $\bar{pf}$ and $c^{O_{b}}$ contains the constant terms that ensure that the perpendicular line passes at the point *p* that is closest to $P_{Q}$.

**Fig 10:**
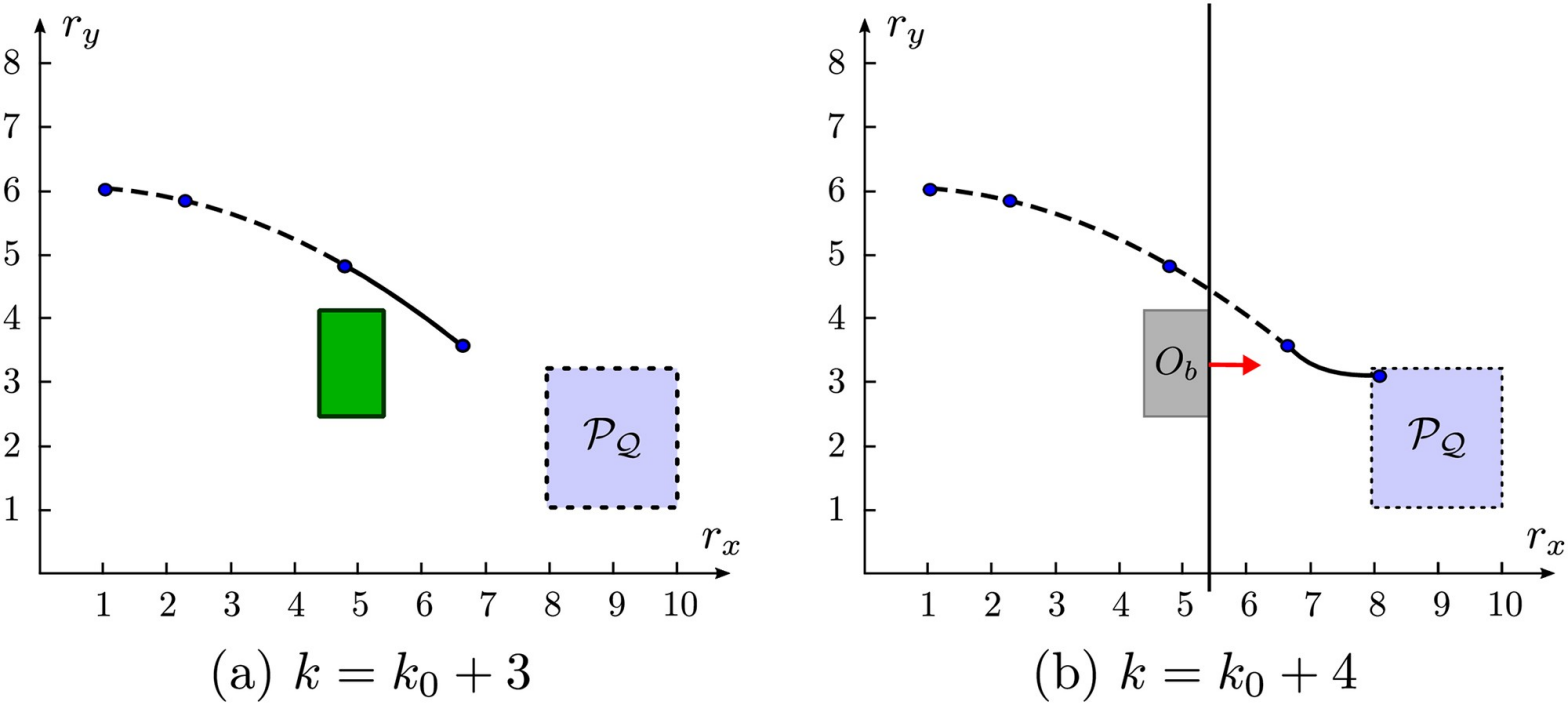
Bygone obstacle rebuttal concept. (a): All the predicted positions of the trajectory lie closer to $P_{Q}$ than the obstacle itself. (b): The straight-line perpendicular to the line that produces the minimum distance between *R*_*f*_ and *O*_*b*_ is represented in *black* and the hard constraint that replaces the bygone obstacle in the subsequent trajectory is depicted as a red arrow.

The illustrative example in Fig 10 represents the inherent concept to this strategy. Each figure depicts in continuous lines concomitantly both the *current* and the *succeeding* positions of the agent and the scheme exhibits the situation *after* all the movement updates in each step.

**Fig 11:**
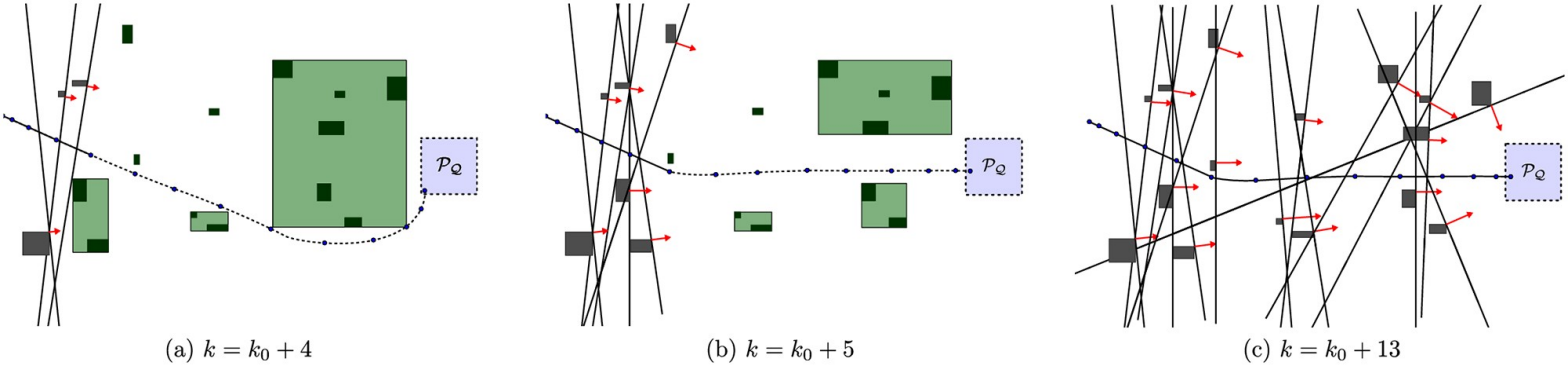
Scheme of the bygone obstacles rebuttal strategy. The projection of each *bygone* obstacle into the position space is represented in gray and its corresponding active *hard* constraint is drawn as a red arrow. In each figure, there are respectively: (a): 3 bygone obstacles. (b): 6 bygone obstacles. (c): 16 bygone obstacles.

In Fig 11 there is a mixed representation of this approach with the Close obstacles Clustering strategy in three different agent positions, as the agent approaches the target. For clarity purposes, we omit both the clustering regions and the clustering distances.

It is important to note that this approach only considers an obstacle as *bygone* if the predicted agent-target distances are smaller than the obstacle-target ones in *all* future steps. This policy works then by reducing the search space through the gradual removal of obstacles at the edges of the environment.

**Alg 6. Bygone Obstacles Rebuttal**.

**Input**: $R_{ob},N_{ob},P_{Q},r\left[k\right],\ldots ,r\left[k+N_{s}-1\right]$

**Output**: *R*_*ob*_

1: **for each** *o* ≤ *N*_*ob*_ **do**

2:  *O*_*b*_ ← *Get Coordinates*(*R*_*ob*_, *o*)

3:  **if** $\max[dist\left(r\left[k\right],P_{Q}\right),\ldots ,dist\left(r\left[k+N_{s}-1\right],P_{Q}\right)]<dist\left(O_{b},P_{Q}\right)$ **then**

4:   $\left(p,f\right)\leftarrow \min_{p\in O_{b},f\in P_{Q}}{∥p-f∥}_{2}$

5:   $Determine\;a\;Straight-line\;Perpendicular\;to\;\bar{pf}$

6:   *coefficients* ← *Translate This Line to Pass at p*

7:   *Add Hard Constraint*(*coefficients*)

8:   *R*_*ob*_ ← *Remove* (*R*_*ob*_, *o*)

9:   *N*_*ob*_ ← *N*_*ob*_ − 1

10:   *o* ← *o* − 1

11:  **end if**

12: **end for**

We represent the algorithm that underlies this strategy in Alg 6, where $dist(r\left[k\right],P_{Q})$ returns the euclidean distance between *r*[*k*] and $P_{Q}$. In case of an obstacle rebuttal, the coordinates of its projection are removed from *R*_*ob*_ and both *N*_*ob*_ and *o* must be decremented, so that the number of obstacles and the subsequent stacked obstacle are correctly evaluated in the next iteration. The inclusion of this algorithm in the planning process is highlighted in blue in Alg 7. Through the assurance that the agent has moved away from the obstacle and will do so in *all* the predicted trajectory, it is possible to rebut bygone obstacles while ensuring the existence of a feasible solution to the optimization problem at the next sample times.

**Alg 7. Closed-loop receding horizon maneuvering with Bygone Obstacles Rebuttal Strategy**.

**Input**: *x*[*k*], *R*_*ob*_, *R*_*f*_

**Output**: *x*[*k* + 1]

1: *Load Simulation Parameters*

2: **while** x[k]∉Q **do**

3:  *R*_*ob*_ ← *Rebut Bygone Obstacles*(*R*_*ob*_, *R*_*f*_, *k*)

4:  *R*_*k*_ ← *Cluster Obstacles in Neighborhood*(*r*[*k*], *R*_*ob*_, *d*_*c*_, *R*_*ic*_, *R*_*sc*_, *R*_*oc*_)

5:  [**A**, **B**]←*Assemble Problem Matrices*(*R*_*k*_, *N*_*s*_)

6:  *u*[*k*]← *Solve MIP Problem*(**A**, **B**)

7:  *x*[*k* + 1]←**A** *x*[*k*]+ **B** *u*[*k*]

8:  *k* ← *k* + 1

9: **end while**

**Fig 12:**
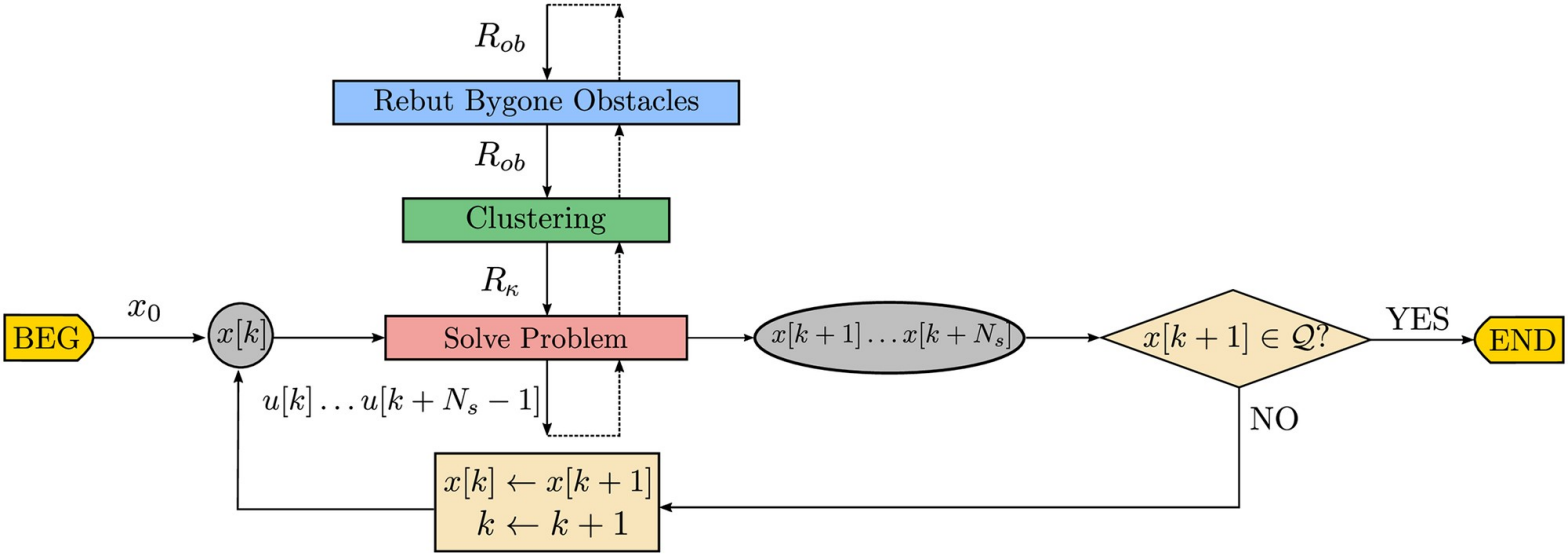
Data flowchart of the closed-loop receding horizon maneuvering with bygone obstacles rebuttal algorithm.

The flowchart in Fig 12 represents the data produced throughout the main steps of the Bygone obstacles rebuttal algorithm. To the previous Clustering algorithm of Fig 9 we prepend a bygone obstacle rebuttal step, represented in *blue*, which removes the bygone obstacles and adds the respective *hard* constraints to the problem to be solved thenceforth.

This strategy replaces the initial *N*_*f*_+ 1 *mixed-integer* avoidance constraints of each bygone obstacle with *only one hard* constraint, and allows for removal *N*_*s*_ *N*_*f*_ binary variables for each bygone obstacle that is replaced by a simple inequality.

### 4.2 Exterior obstacles contempt strategy

**Fig 13:**
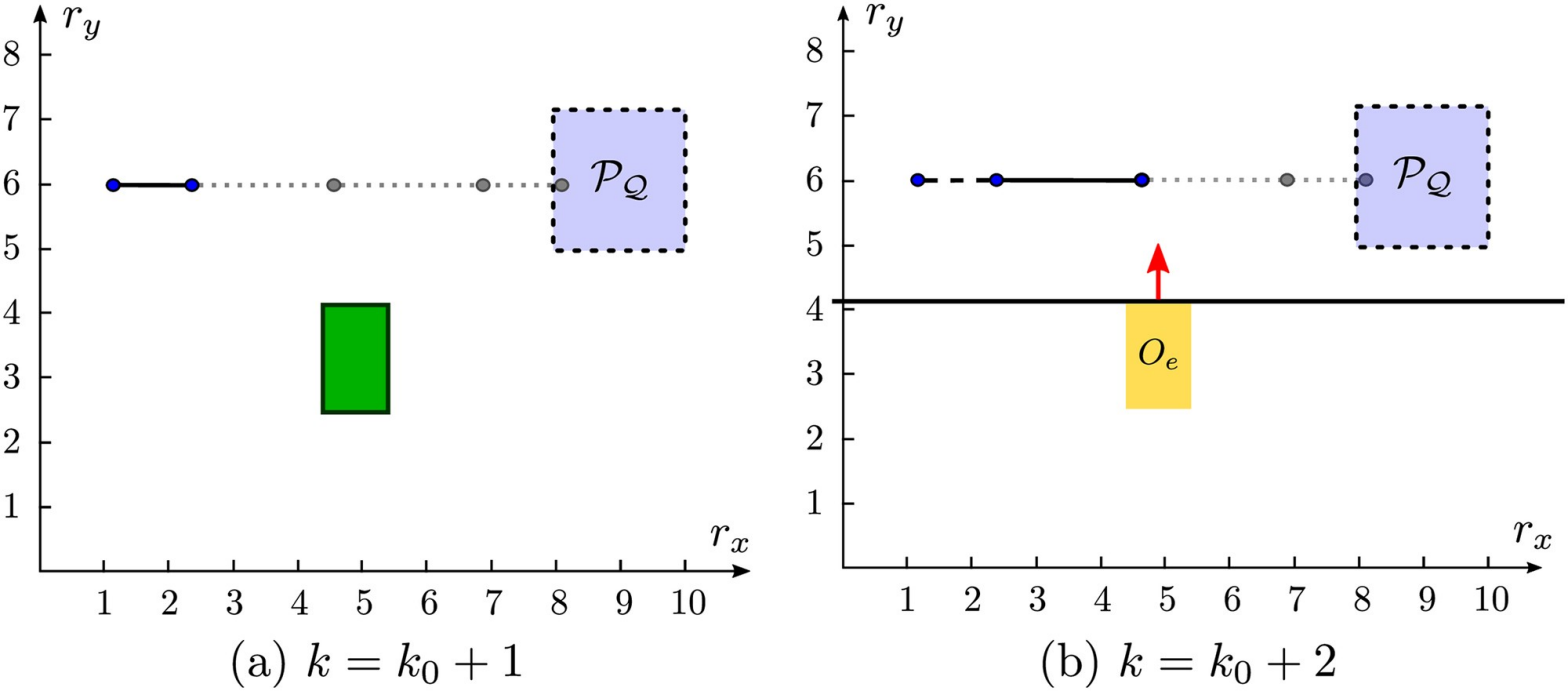
Exterior obstacle contempt concept. (a): After the first step, all predicted positions represented in gray lie above the upper face of the obstacle. (b): It is possible to replace this exterior obstacle in the subsequent trajectory by the hard constraint which corresponds to the red arrow that is drawn from the side of *O*_*e*_ that is collateral to the entire trajectory.

Once the agent obtains a path to the target $P_{Q}$, if *all* the *predicted* positions of the agent lay on the *same side* of some obstacle whose projection into the position space is given by O_*e*_, then such obstacle may be replaced by the hard constraint corresponding to this very side. The example in Fig 13 illustrates this idea.

It is worth emphasizing that, while in Eq (9) *f* ranges from 1 to *N*_*f*_, in the case of the exterior obstacle of Eq (16), $\tilde{f}$ assumes a single value, which is that of the constraint that is always obeyed along the horizon.

$$\begin{array}{c} b_{\tilde{f}}^{O_{e}}x\left[k+j|k\right]\geq c_{\tilde{f}}^{O_{e}},\forall j\in \left\{1,2,\ldots ,N_{s}\right\} \end{array}$$ (16)

A simple check upon the correspondence of the values that the binary avoidance variables assume for a given obstacle side along the complete prediction horizon would represent a *sufficient* condition for identifying an obstacle as exterior. However, there are cases in which an obstacle can be exterior and *not* have all binary variables fixed True to a certain side throughout the whole prediction horizon, due to the redundancy resulting from the overlap of valid regions in the presence of a polygonal obstacle.

For example, at *k* = *k*_0_+ 1 in Fig 13a, if the solver sets the variable of the valid region to the *left*-hand side of the obstacle instead of the one *above* it, the obstacle would not be identified as exterior at *k* = *k*_0_+ 2 in Fig 13b. In such a case, there are obstacles that could be removed at a certain instant, but would not. In fact, we do *not* rely only on the binaries to verify the obstacle contempt condition, but in fact we calculate whether the condition of the constraint is respected for each *cluster* by the predicted positions of the agent at every instant. The choice of which constraint to adopt in the case of the overlap of more than one valid region becomes then a design decision. Thus, as the agent circumvents a cluster and proceeds in its path to the target, the obstacles inside it become exterior and a single hard constraint is added replacing the whole cluster.

**Fig 14:**
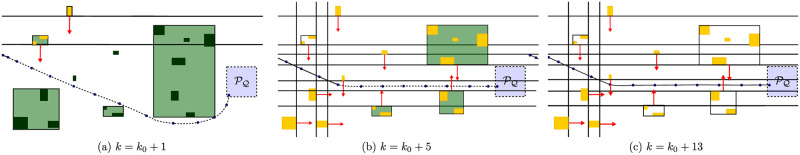
Scheme of the exterior obstacles clustering strategy. The *exterior* obstacles are drawn in yellow and the respective *hard* constraints are represented by red arrows. (a): 7 clusters are identified, 2 of which contain 3 obstacles that become exterior. (b): 3 clusters which contain the 8 remaining obstacles are identified as exterior. (c): Simulation ends with the 16 obstacles replaced by 10 *hard* constraints.

In Fig 14 there is a mixed representation of this approach with the Close Obstacles Clustering strategy in three different agent positions.

**Alg 8. Exterior Obstacles Identification**.

**Input**: *U*, *R*_*κ*_, *N*_*c*_, *N*_*s*_

**Output**: *ext*, *current*

1: *current*[1: *N*_*s*_]←*Get Trajectory Prediction*(*U*)

2: *max*_*r*_*x*_ ← *max*(*Load r*_*x*_ *Coordinates*(*current*))

3: *max*_*r*_*y*_ ← *max*(*Load r*_*y*_ *Coordinates*(*current*))

4: *min*_*r*_*x*_ ← *min*(*Load r*_*x*_ *Coordinates*(*current*))

5: *min*_*r*_*y*_ ← *min*(*Load r*_*y*_ *Coordinates*(*current*))

6: *c* ← 1

7: **while** *c* ≤ *N*_*c*_ **do**

8:  *isLefthand*, *isBelow*, *isRighthand*, *isAbove* ← False

9:  **if** *max*_*r*_*x*_ < *Get Left* − *hand Side Coordinate*(*R*_*κ*_, *c*) **then**

10:   *isLefthandSide* ← True

11:  **else if** *max*_*r*_*y*_ < *Get Lower Coordinate*(*R*_*κ*_, *c*) **then**

12:   *isBelow* ← True

13:  **else if** −*min*_*r*_*x*_ < −*Get Right* − *handSide Coordinate*(*R*_*κ*_, *c*) **then**

14:   *isRighthandSide* ← True

15:  **else if** −*min*_*r*_*y*_ < −*Get Upper Coordinate*(*R*_*κ*_, *c*) **then**

16:   *isAbove* ← True

17:  **end if**

18:  *ext*[*c*]←*isLefthandSide* ∨ *isBelow* ∨ *isRighthandSide* ∨ *isAbove*

19:  *c* ← *c* + 1

20: **end while**

**Alg 9. Exterior Obstacles Contempt**.

**Input**: *R*_*ob*_, *N*_*ob*_, *R*_*κ*_, *N*_*c*_, *ext*, *current*

**Output**: *R*_*ob*_, *N*_*ob*_

1: *c* ← 1

2: **while** *c* ≤ *N*_*c*_ **do**

3:  **if** *ext*[*c*] **then**

4:   *coefficients* ← *Get Active Exterior Constraint*(*c*, *R*_*κ*_, *current*)

5:   *Add Hard Constraint*(*coefficients*)

6:   *o* ← 1

7:   **while** *o* ≤ *N*_*ob*_ **do**

8:    **if** *Is Obstacle Inside Cluster*(*o*, *c*, *R*_*ob*_, *R*_*c*_) **then**

9:     *R*_*ob*_ ← *Remove* (*R*_*ob*_, *o*)

10:     *N*_*ob*_ ← *N*_*ob*_ − 1

11:     *o* ← *o* − 1

12:    **end if**

13:    *o* ← *o* + 1

14:   **end while**

15:  **end if**

16:  *c* ← *c* + 1

17: **end while**

The pseudocode that identifies exterior obstacles is represented in Alg 8. It consists basically in the comparison of the sides of each cluster, that were previously identified in the clustering step, with the most extreme predicted trajectory coordinates, either at the left-hand side, below, at the right-hand side or above, where ∨ is the boolean OR operator. Here, *ext* is an array of boolean variables, in which *ext*[*c*] = True if cluster *c* is exterior. Then, to replace the exterior obstacle it is enough to add the hard constraint that corresponds to the new exterior cluster in the planning problem to be solved thenceforth, with the proper contempt of the obstacles that belong to this cluster from the obstacle set, as Alg 9 shows. Here, function *Get Active Exterior Constraint*() returns the coefficients of the exterior constraint that replaces the exterior obstacle.

**Alg 10. Closed-loop receding horizon maneuvering with Exterior Obstacles Contempt Algorithm**.

**Input**: *x*[*k*], *R*_*ob*_, *R*_*f*_

**Output**: *x*[*k* + 1]

1: *Load Simulation Parameters*

2: **while** x[k]∉Q **do**

3:  *R*_*ob*_ ← *Rebut Bygone Obstacles*(*R*_*ob*_, *R*_*f*_, *k*)

4:  *R*_*k*_ ← *Cluster Obstacles in Neighborhood*(*r*[*k*], *R*_*ob*_, *d*_*c*_, *R*_*ic*_, *R*_*sc*_, *R*_*oc*_)

5:  [**A**, **B**]←*Assemble Problem Matrices*(*R*_*k*_, *N*_*s*_)

6:  *u*[*k*]← *Solve MIP Problem*(**A**, **B**)

7:  *R*_*ob*_ ← *Contempt Exterior Obstacles*(*R*_*ob*_, *u*[*k*], …, *u*[*k* + *N*_*s*_ − 1])

8:  *x*[*k* + 1]←**A** *x*[*k*]+ **B** *u*[*k*]

9:  *k* ← *k* + 1

10: **end while**

**Fig 15:**
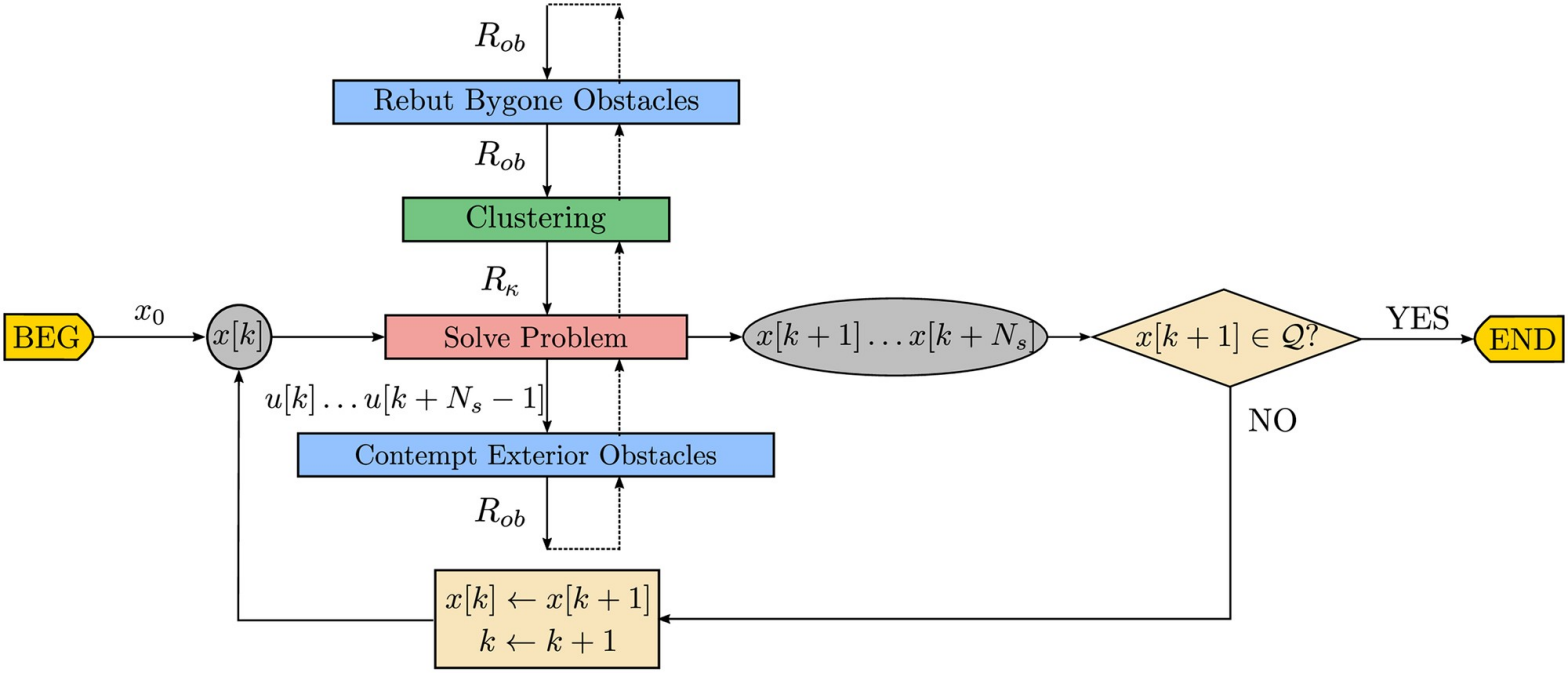
Data flowchart of the closed-loop receding horizon maneuvering with exterior obstacles contempt algorithm. To the previous Bygone obstacles rebuttal algorithm of Fig 12, we append an exterior obstacle contempt step, represented in *blue*, which replaces the exterior obstacles by the respective *hard* constraints to the problem to be solved from then on.

We represent the pseudocode of this strategy in Alg 10, with the contempt of exterior obstacles highlighted in *blue*, while the flowchart in Fig 15 represents the data produced throughout the main steps of the Exterior obstacles contempt algorithm.

Once a cluster is identified as exterior, this strategy replaces the initial *N*_*f*_+ 1 *mixed-integer* avoidance constraints of each exterior cluster with *only one hard* constraint for the whole cluster, and allows for removal *N*_*s*_ *N*_*f*_ binary variables for each cluster that is replaced by a simple inequality.

### 4.3 Iterative clustering distance tuning

To decrease the number of binary variables in the optimization problem, the Close obstacles clustering strategy may entail the elimination of spaces initially available for navigation. This, in turn, might lead to an infeasible optimization problem, even when in the absence of clustering a feasible trajectory could be determined. In this section we will describe a scheme that circumvents this problem and allows to obtain a feasible trajectory to the final region, when one exists.

To the previous cluster avoidance algorithm in Alg 10 we append an iterative deepening search in the clustering avoidance subproblems, with a *non-increasing* clustering distance highlighted in blue. In Alg 11, if we initialize the clustering distance *d*_*c*_ with a value large enough, the problem could be *infeasible*. As a result, the if condition of line 7 would hold value True, *d*_*c*_ would be reduced according to the expression in Eq (17) and the continue statement of line 9 would break the loop execution. As the clustering distance is held as a global variable, in the next loop iteration, the algorithm would make its prediction with the *updated* value, and the execution would continue until the algorithm was able to find a feasible path to the target set. On the other hand, if the value of *d*_*c*_ did not produce initially an infeasible problem, then a trajectory to the final region would have already been found.

$$\begin{array}{c} d_{c}\leftarrow s_{r}d_{c}. \end{array}$$ (17)

Here, 0 < *s*_*r*_ < 1 is taken as a shrinking rate for *d*_*c*_. Small values of *s*_*r*_ imply a high cutback on *d*_*c*_ and a smaller number of clustering attempts until the strategy finds a viable path to Q, potentially in a configuration with many clusters in the environment. On the other hand, high values of *s*_*r*_ entail a gradual reduction on *d*_*c*_ and allow the attainment of a cluster configuration that is closer to the minimal number of clusters, possibly at the expense of a greater number of infeasible solution trials.

**Alg 11. Closed-loop receding horizon maneuvering with the Iterative Clustering Distance Tuning Algorithm**.

**Input**: *x*[*k*], *R*_*ob*_, *R*_*f*_

**Output**: *x*[*k* + 1]

1: *Load Simulation Parameters*

2: **while** x[k]∉Q **do**

3:  *R*_*ob*_ ← *Rebut Bygone Obstacles*(*R*_*ob*_, *R*_*f*_, *k*)

4:  *R*_*k*_ ← *Cluster Obstacles in Neighborhood*(*r*[*k*], *R*_*ob*_, *d*_*c*_, *R*_*ic*_, *R*_*sc*_, *R*_*oc*_)

5:  [**A**, **B**]←*Assemble Problem Matrices*(*R*_*k*_, *N*_*s*_)

6:  *u*[*k*]← *Solve MIP Problem*(**A**, **B**)

7:  **if** *Is Problem Infeasible*(*u*[*k*]) **then**

8:   *d*_*c*_ ← *Shrink Clustering Distance*(*d*_*c*_)

9:   *continue*

10:  **end if**

11:  *R*_*ob*_ ← *Contempt Exterior Obstacles*(*R*_*ob*_, *u*[*k*], …, *u*[*k* + *N*_*s*_ − 1])

12:  *x*[*k* + 1]←**A** *x*[*k*]+ **B** *u*[*k*]

13:  *k* ← *k* + 1

14: **end while**

**Fig 16:**
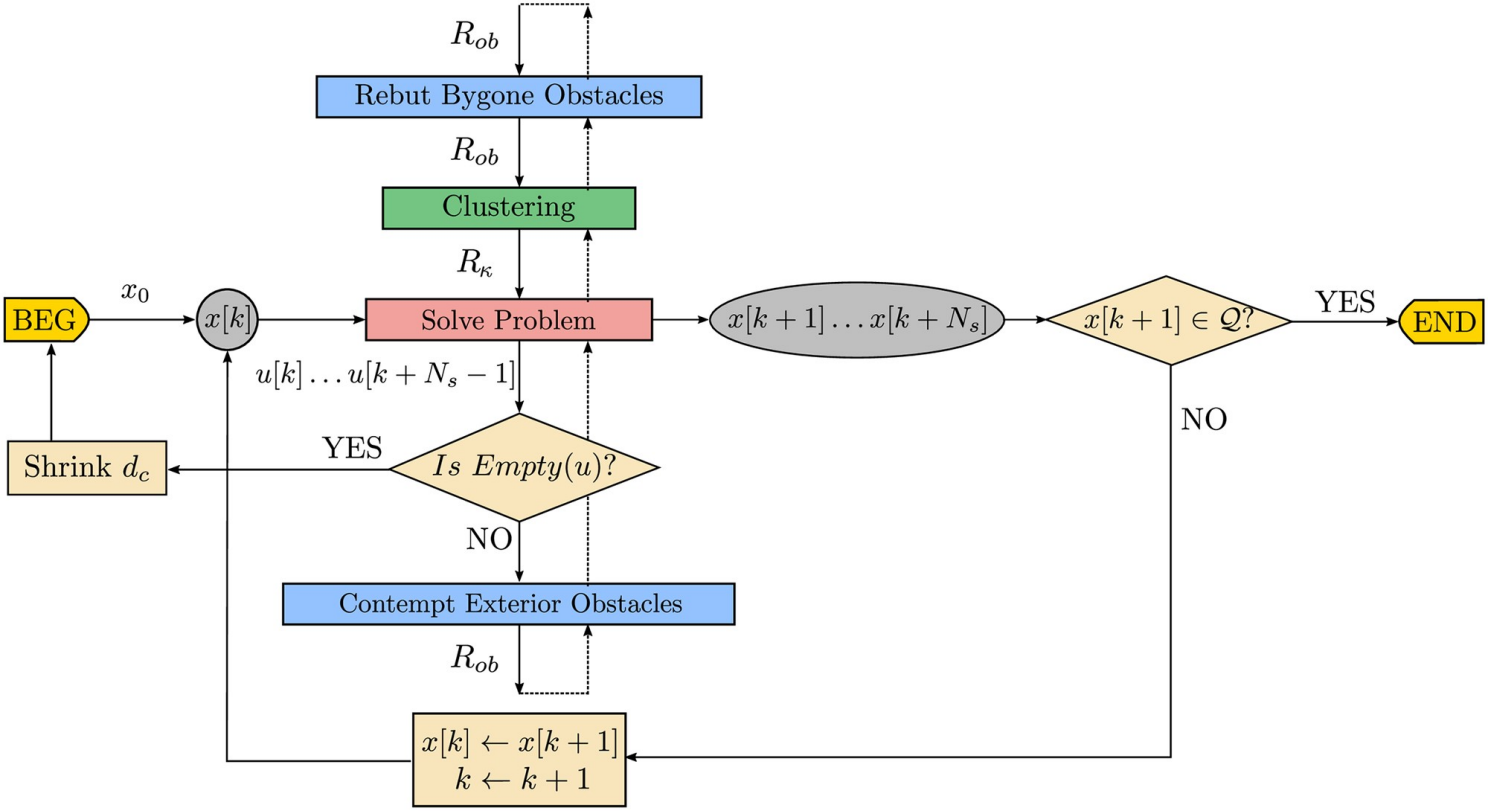
Data flowchart of the closed-loop receding horizon maneuvering with iterative clustering distance tuning algorithm.

The flowchart in Fig 16 represents the data produced throughout the main steps of the Iterative clustering algorithm. To the previous Exterior clustering flowchart of Fig 15 we add the continue statement, represented in *blue*, which decreases the clustering distance in the avoidance subproblem to be solved thenceforth.

## 5 Simulation scenarios

The agent model is that of a particle [14, 34, 37] moving in a plane with axes *r*_*x*_ and *r*_*y*_ orthogonal to each other, and we define the position vector as **r**^*T*^ = [*r*_*x*_ *r*_*y*_]. The inputs are the accelerations *a*_*x*_ and *a*_*y*_, which result in velocities *v*_*x*_ and *v*_*y*_ aligned respectively with the *r*_*x*_ and *r*_*y*_ axes. In state-space, the continuous-time model is $\dot{x}=A_{c}x+B_{c}u$, with state vector **x**^*T*^ = [*r*_*x*_ *v*_*x*_ *r*_*y*_ *v*_*y*_] and control vector **u**^*T*^ = [*a*_*x*_ *a*_*y*_]. Then, matrices *A*_*c*_ and *B*_*c*_ are given by Eqs (18) and (19).

$$\begin{array}{c} A_{c}=[\begin{array}{cccc} 0 & 1 & 0 & 0\\ 0 & 0 & 0 & 0\\ 0 & 0 & 0 & 1\\ 0 & 0 & 0 & 0 \end{array}], \end{array}$$ (18)

$$\begin{array}{c} B_{c}=[\begin{array}{cc} 0 & 0\\ 1 & 0\\ 0 & 0\\ 0 & 1 \end{array}]. \end{array}$$ (19)

The plant model must be discretized to be used as the internal controller model, since the MPC controller is implemented in discrete-time. Then, by Zero-Order Hold discretization [46] we obtain a model of the form **x**[*k* + 1] = **A** **x**[*k*]+ **B** **u**[*k*]. For a sample period of 0.8 time units, the matrices **A** and **B** are then given by Eqs (20) and (21).

Here we assume the units are: *m* for length and *s* for time.

$$\begin{array}{c} A=[\begin{array}{cccc} 1 & 0.8 & 0 & 0\\ 0 & 1 & 0 & 0\\ 0 & 0 & 1 & 0.8\\ 0 & 0 & 0 & 1 \end{array}]\;, \end{array}$$ (20)

$$\begin{array}{c} B=[\begin{array}{cc} 0.32 & 0\\ 0.8 & 0\\ 0 & 0.32\\ 0 & 0.8 \end{array}]\;. \end{array}$$ (21)

The following parameters are adopted in the controller settings of the agent:

1. constraints over velocities and accelerations: −10 *m*/*s* ≤ *v*_*x*_, *v*_*y*_ ≤ 10 *m*/*s* and −3 *m*/*s*^2^ ≤ *a*_*x*_, *a*_*y*_ ≤ 3 *m*/*s*^2^
2. maximal horizon: *N*_*s*_ = 18
3. constraints over terminal velocities: −5 × 10^−3^ *m*/*s* ≤ *v*_*x*_, *v*_*y*_ ≤ 5 × 10^−3^ *m*/*s*
4. weight of the sum of the absolute values of the accelerations: *γ* = 1

The weight *γ* was chosen to adjust a compromise between fuel expense and time minimization, so that the choice of *γ* makes both contributions comparable in the cost function.

**Fig 17:**
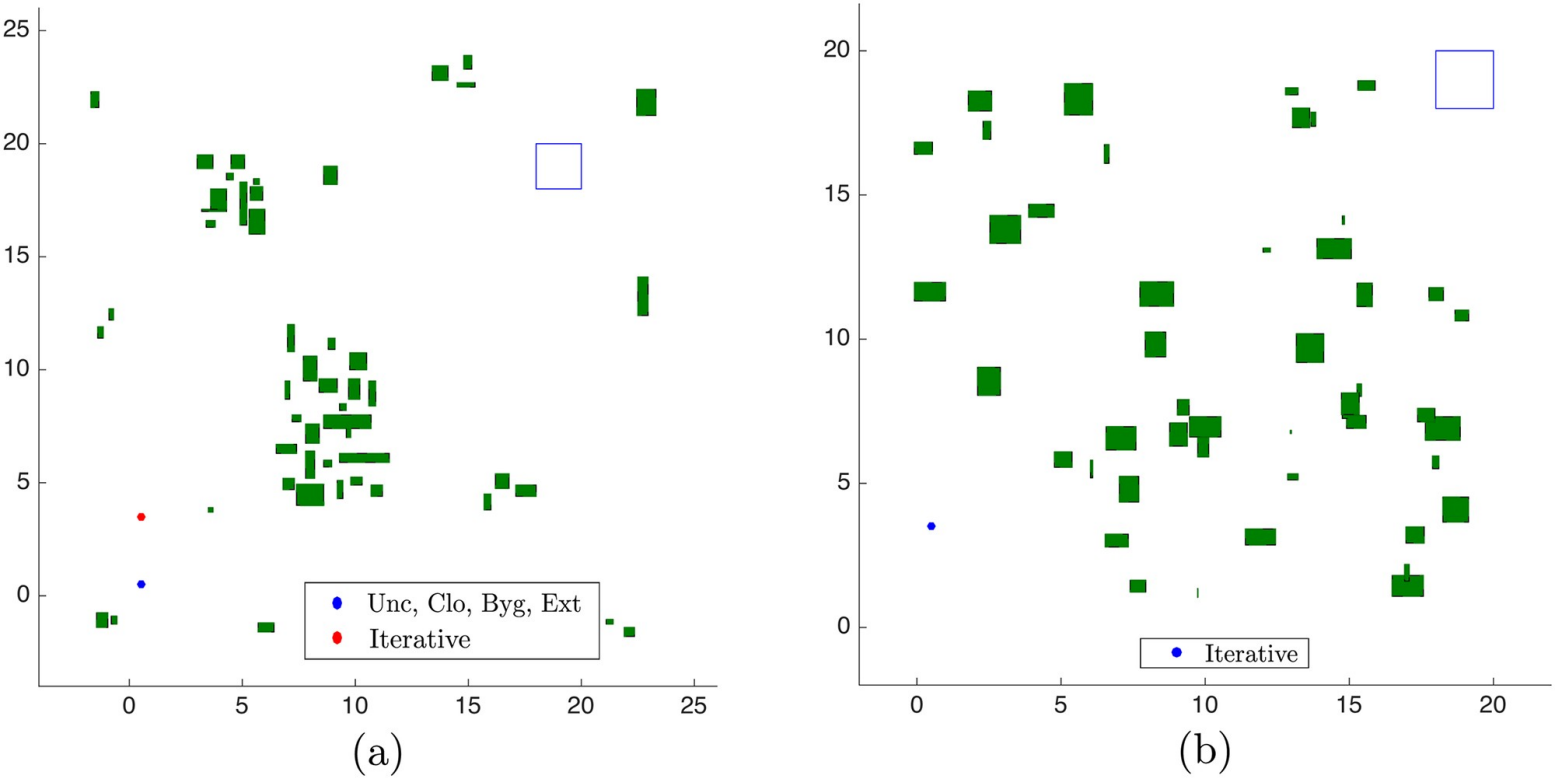
Maps used for trajectory planning. (a) Map used for the Unclustered, Clustering, Bygone, Exterior and Iterative strategies. (b) Random Map used for the Iterative strategy.

The initial state and terminal set that we use here are summarized in Table 1 for each clustering strategy. The agent starts at rest and is required to reach the terminal set with low speed. Representative maps are shown in Fig 17a, with the *blue* dot representing the initial condition of the Unclustered, Clustering, Bygone and Exterior strategies and the *red* dot for the initial position of the Iterative strategy, while in Fig 17b we represent the random map used. In the Iterative strategy simulations, the agent was brought closer to the central obstacles to increase the chances of obtaining initially an infeasible problem.

**Table 1:** Experiments input data.

Strategy	x_0_	Q	*d*_*c*_	*R*_*c*_	*N*_*s*_	*T*[*s*]
Unclustered	[0.5 0 0.5 0]^*T*^	|*v*_*x*_|, |*v*_*y*_|≤5 × 10^−3^ *m*/*s*, 18 ≤ *r*_*x*_, *r*_*y*_ ≤ 20	–	–	18	0.8
Close obstacles	[0.5 0 0.5 0]^*T*^	|*v*_*x*_|, |*v*_*y*_|≤5 × 10^−3^ *m*/*s*, 18 ≤ *r*_*x*_, *r*_*y*_ ≤ 20	[1.5 2.5 3.5]^*T*^	[4 8 ∞]^*T*^	18	0.8
Bygone obstacles	[0.5 0 0.5 0]^*T*^	|*v*_*x*_|, |*v*_*y*_|≤5 × 10^−3^ *m*/*s*, 18 ≤ *r*_*x*_, *r*_*y*_ ≤ 20	[1.5 2.5 3.5]^*T*^	[4 8 ∞]^*T*^	18	0.8
Exterior obstacles	[0.5 0 0.5 0]^*T*^	|*v*_*x*_|, |*v*_*y*_|≤5 × 10^−3^ *m*/*s*, 18 ≤ *r*_*x*_, *r*_*y*_ ≤ 20	[1.5 2.5 3.5]^*T*^	[4 8 ∞]^*T*^	18	0.8
Iterative	[0.5 0 3.5 0]^*T*^	|*v*_*x*_|, |*v*_*y*_|≤5 × 10^−3^ *m*/*s*, 18 ≤ *r*_*x*_, *r*_*y*_ ≤ 20	[3 6 9]^*T*^	[4 8 ∞]^*T*^	18	0.8
Iterative (random)	[0.5 0 3.5 0]^*T*^	|*v*_*x*_|, |*v*_*y*_|≤5 × 10^−3^ *m*/*s*, 18 ≤ *r*_*x*_, *r*_*y*_ ≤ 20	[3 6 9]^*T*^	[4 8 ∞]^*T*^	18	0.8

Initial State **x**_**0**_, Terminal Set Q, Clustering Distance *d*_*c*_, Clustering Radius *R*_*c*_, Number of Steps *N*_*s*_ and Sampling Period *T* for experiments of the Unclustered, Clustering, Bygone, Exterior and Iterative strategies

In order to engender a reference to the performance of the algorithms that this work proposes, we employ a version of the problem with the obstacle avoidance constraints relaxed, as a *benchmark* to the simulation times. Such version, denominated as *Relaxed* from now on, is achieved by changing to 0 the right-side of Eq (9b), what allows, as a valid solution state, none of the avoidance variables to be activated at every time step.

Also recall that, as a simplification, we approximate each obstacle by its rectangular envelope with sides parallel to the axes. If necessary, [34] provides a more general formulation. Finally, the CPLEX toolbox from IBM ILOG was used for solving the MILP problem in Matlab environment.

### 5.1 Random obstacles generation

Consider the uniformly distributed variables *b*_*x*_, *b*_*y*_, *h*_*x*_, *h*_*y*_, where *b*_*x*_ and *b*_*y*_ are both limited between 0 and 1 and represent relative coordinates of the obstacle center, *h*_*x*_ and *h*_*y*_ are both limited between 0.04 and 1.25 and represent respectively the width and height of the obstacle. Consider also *L* = [*L*_*x*_ *L*_*y*_]^*T*^, in which *L*_*x*_ and *L*_*y*_ are both 20 and correspond to the dimensions of the environment.

Let *f*_0*x*_, *f*_0*y*_, *f*_1*x*_, *f*_1*y*_, *B*_*x*_, *B*_*y*_, *m*_*x*_ and *m*_*y*_ be 8 real numbers, where *f*_0*x*_ = *f*_0*y*_ = 0 and *f*_1*x*_ = *f*_1*y*_ = 20 describe respectively the smallest and largest coordinate values in the *x* and *y* axes within the region that contains them in the environment, *B*_*x*_ and *B*_*y*_ are both limited between 0 and 20 and identify the coordinates of the center of an obstacle and *m*_*x*_ and *m*_*y*_ account for the border around the environment which defines the territory that contains the random obstacle set, where *m*_*x*_ = *m*_*y*_ = 0.

Then, an obstacle in the environment with sides parallel to the axes and center in [*B*_*x*_ *B*_*y*_]^*T*^ can be randomly generated according to the expression in Eq (22),

$$\begin{array}{c} R_{ob}={\left[R_{x0}\;R_{y0}\;R_{x1}\;R_{y1}\right]}^{T}={\left[\begin{array}{c} B_{x}-\frac{w}{2}\;\;B_{y}-\frac{h}{2}\;\;B_{x}+\frac{w}{2}\;\;B_{y}+\frac{h}{2} \end{array}\right]}^{T}, \end{array}$$ (22)

where *B* = (*f*_0_ − *m*)+ (*f*_1_ − *f*_0_+ 2*m*)*b*, in which *B* ∈ {*B*_*x*_, *B*_*y*_}, *b* ∈ {*b*_*x*_, *b*_*y*_}, *m* ∈ {*m*_*x*_, *m*_*y*_}, *f*_0_ ∈ {*f*_0*x*_, *f*_0*y*_} and *f*_1_ ∈ {*f*_1*x*_, *f*_1*y*_}.

For the generation of uniformly distributed pseudorandom numbers, we used the rand function in Matlab environment.

## 6 Results

### 6.1 Unclustered scenario

**Fig 18:**
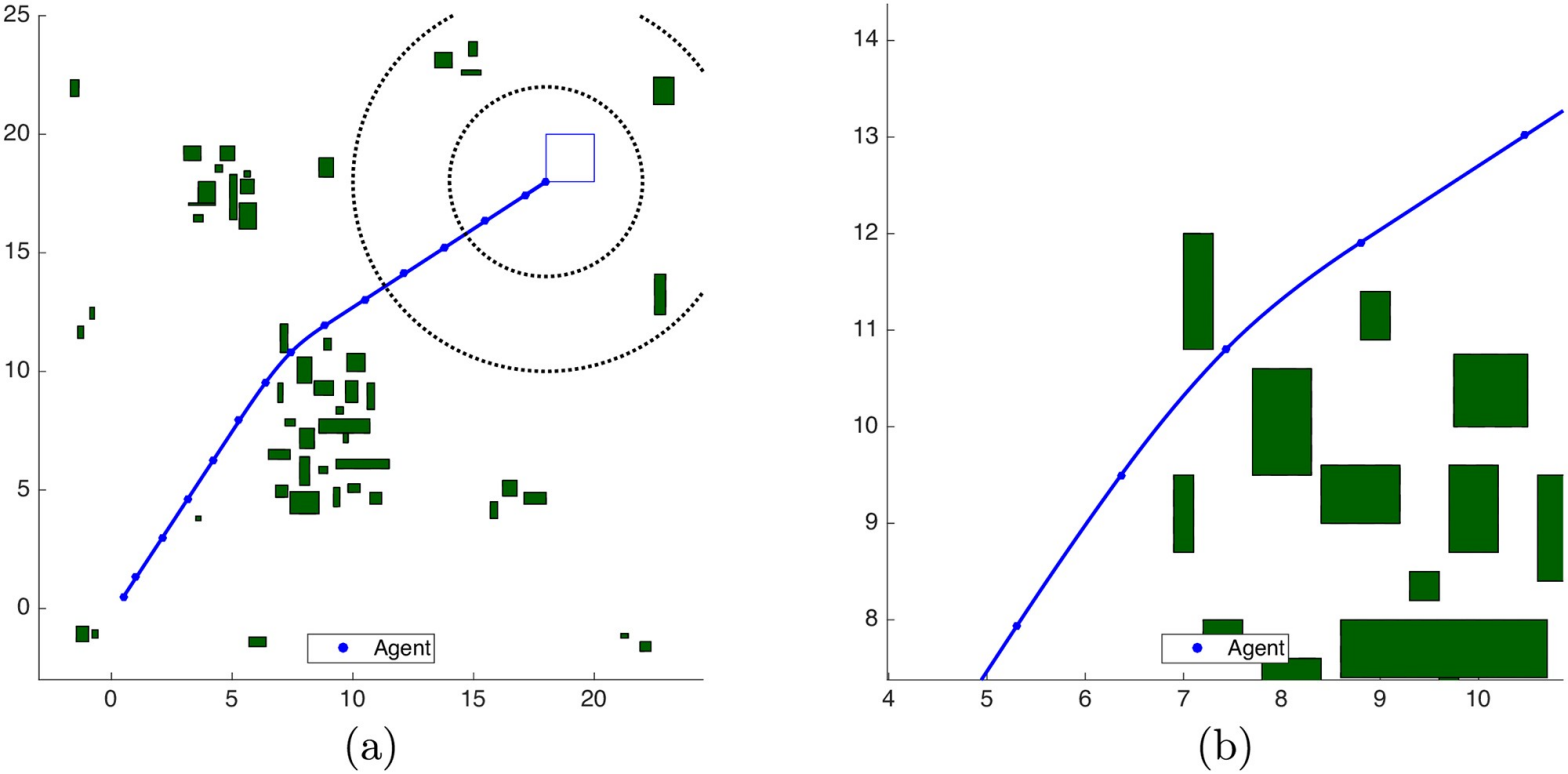
Trajectory of the unclustered strategy. (a): Complete trajectory. (b): Central Obstacles Zoom.

For comparison purposes, we obtained the average time to calculate the *complete* trajectory that results from the obstacle avoidance procedure in an unclustered scenario, as [34] proposes. For the Unclustered, Clustering, Bygone, Exterior and Iterative strategies in maps of Fig 17a and 17b we obtained each of these times as an *average* of 30 simulations. The resulting path for the Unclustered strategy is represented in Fig 18a, with a zoom of the curve drawn among the obstacles in Fig 18b.

**Fig 19:**
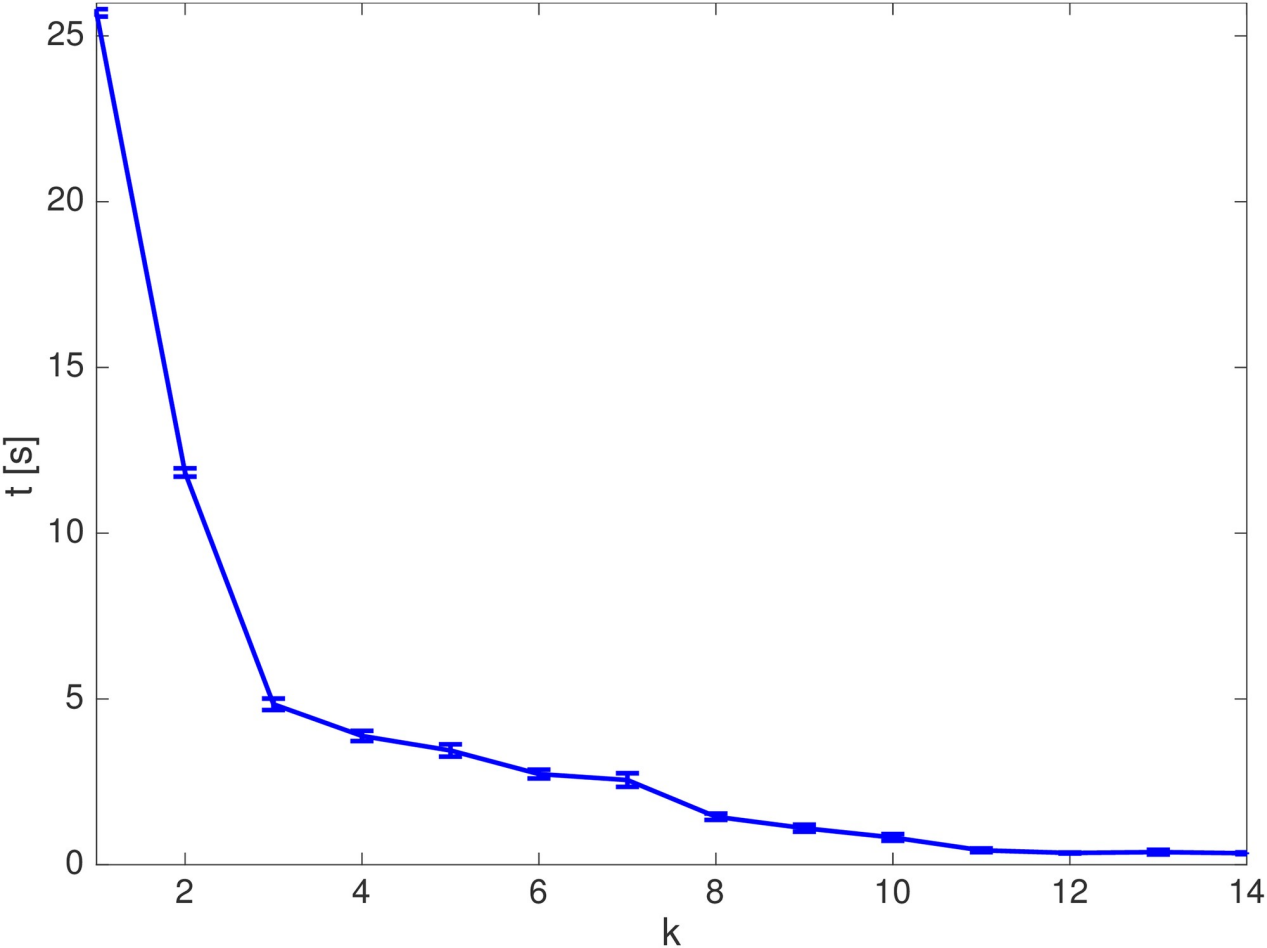
Average elapsed time and standard deviation of the simulations in the unclustered scenario.

The average simulation times along each iteration of Alg 1 are monotonically decreasing, as Fig 19 shows with the respective standard deviation. For the unclustered scenario, the average total simulation time, *i.e*. the average of the summation of *all* optimization times from *k* = 0, 1, …, *N*[*k*] until reaching the target set at *N*[*k*], is *t*_*u*_ ≈ 61.65 *s*.

### 6.2 Close obstacles clustering strategy

**Fig 20:**
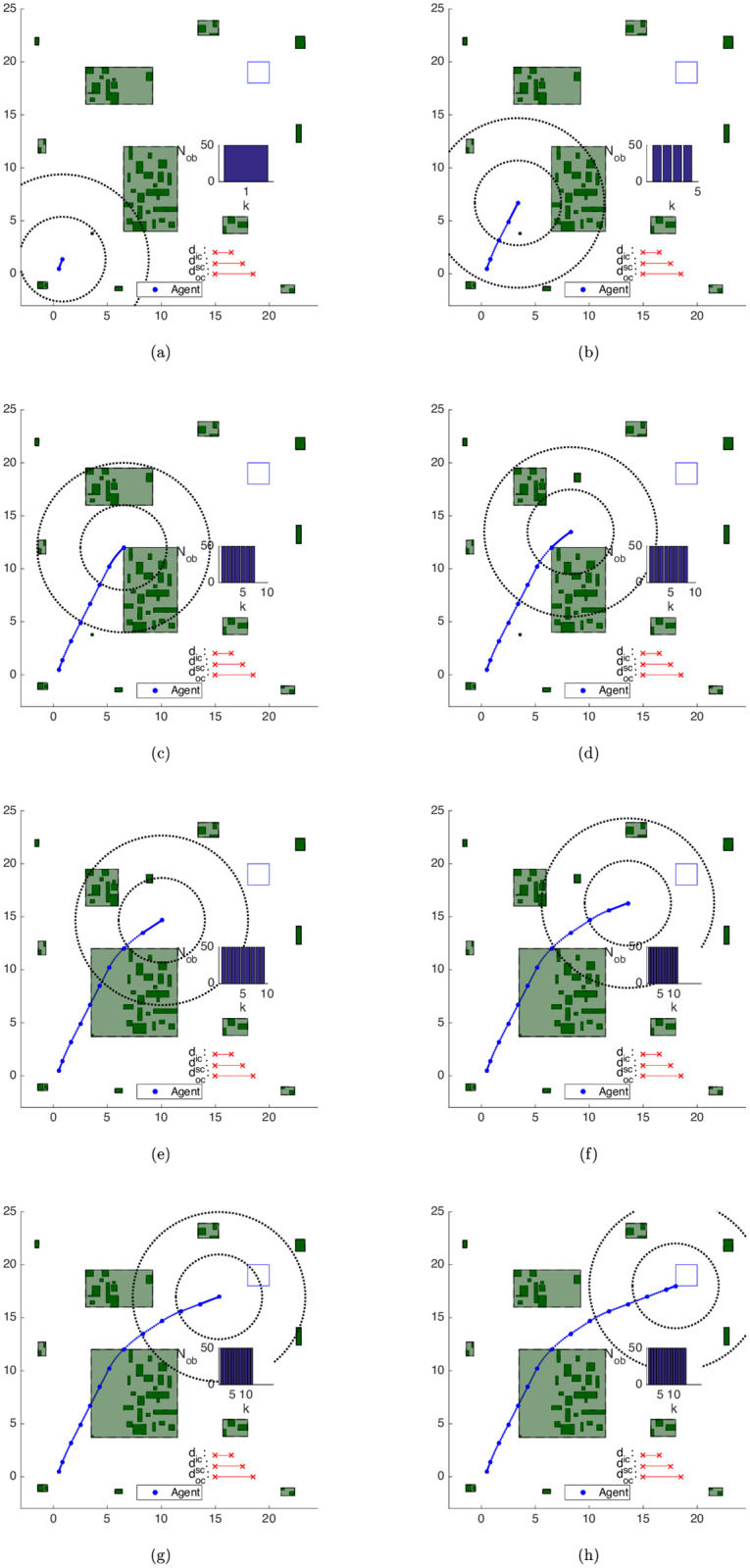
Trajectory of the close obstacles clustering strategy in the map of Fig 17a. (a) Step 1. (b) Step 4. (c) Step 7. (d) Step 8. (e) Step 9. (f) Step 11. (g) Step 12. (h) Step 14.

Algorithm 5 was applied for the results in this subsection. In Fig 20, we can observe the evolution of the clustering strategy as the agent moves towards the goal area. Each figure represents in continuous lines concomitantly both the *current* and the *succeeding* positions of the agent and each scheme exhibits the situation *after* all the movement updates in each step.

Algorithm 3 initially groups the obstacles into 12 clusters, which are depicted in Fig 20a. After the turn to the right-hand side in Fig 20c, the north cluster enters the *inner-zone* and is split up into 2 clusters (Fig 20d). Once there are still some obstacles of the cluster to the south of the agent in the *inner-zone*, the algorithm considers *d*_*ic*_ as the active clustering distance and does not merge the clusters to the south and to the southwest of the territory, totaling 13 clusters in step 8.

In Fig 20e, the clusters to the south and to the southwest of the agent are lumped together, and the same also happens to the clusters to the north and to the northwest of the territory in the following steps (Fig 20f and 20g). Once there is no significant change in the environment, the initially planned trajectory is strictly followed, with $J^{⋆}\left[k+1\right]=\hat{J}\left[k+1|k\right]=J^{⋆}\left[k\right]-\gamma {∥\hat{u}\left[k|k\right]∥}_{1}-1$, where $\hat{J}\left[k+1|k\right]$ is the predicted solution cost for time *k* + 1 given *k*, *J*^⋆^[*k* + 1] is the optimal cost at time *k* + 1 and $∥\hat{u}{\left[k|k\right]∥}_{1}$ is the ℓ_1_-norm of the control effort applied at time step *k*.

In this approach, the obstacle set is used only as input of the clustering procedure, without any update to its components. This means that the computational load remains approximately fixed throughout the maneuver, except for the natural clustering variations due to the change in the relative position between the agent and the obstacles. This is evident in the representation of the number of obstacles *N*_*ob*_ at the *right* of every plot in Fig 20a–20h.

**Fig 21:**
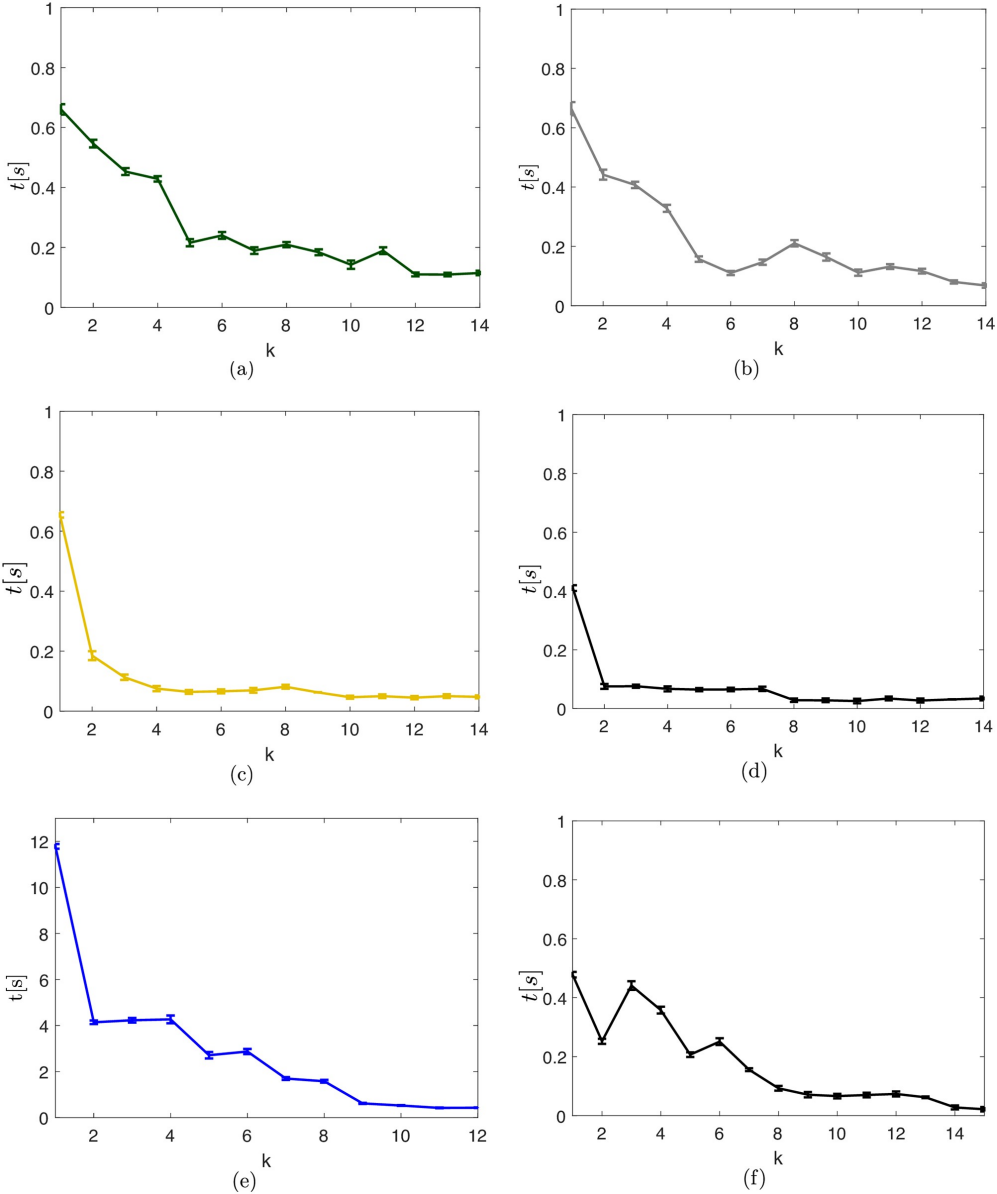
Average simulation times with standard deviation. (a): Clustering strategy in the map of Fig 17a. (b): Bygone obstacles clustering strategy in the map of Fig 17a. (c): Exterior obstacles clustering strategy in the map of Fig 17a. (d): Iterative clustering strategy in the map of Fig 17a. (e): Unclustered strategy in the map of Fig 17b. (f): Iterative clustering strategy in the map of Fig 17b.

With the Clustering strategy, Fig 21a shows the average time spent on each algorithm iteration. The clustering procedure has as main consequence, regarding the computational times along each algorithm iteration, an expressive cutback when compared to the ones of the Unclustered case in Fig 19. By clustering two close obstacles, we remove the need for additional branches made by the branch-and-bound algorithm along the path of an agent that circumvents these obstacles.

The average clustering time along the whole trajectory of the agent is $t_{K,k}\approx 0.18\;s$. The average total simulation time, *i.e*. the average of the summation of the times of *all* optimizations realized until the target set was reached is *t*_*c*_ ≈ 3.79 *s*. The Clustering strategy has an average speedup of 16.26 regarding the Unclustered strategy.

### 6.3 Bygone obstacles rebuttal strategy

**Fig 22:**
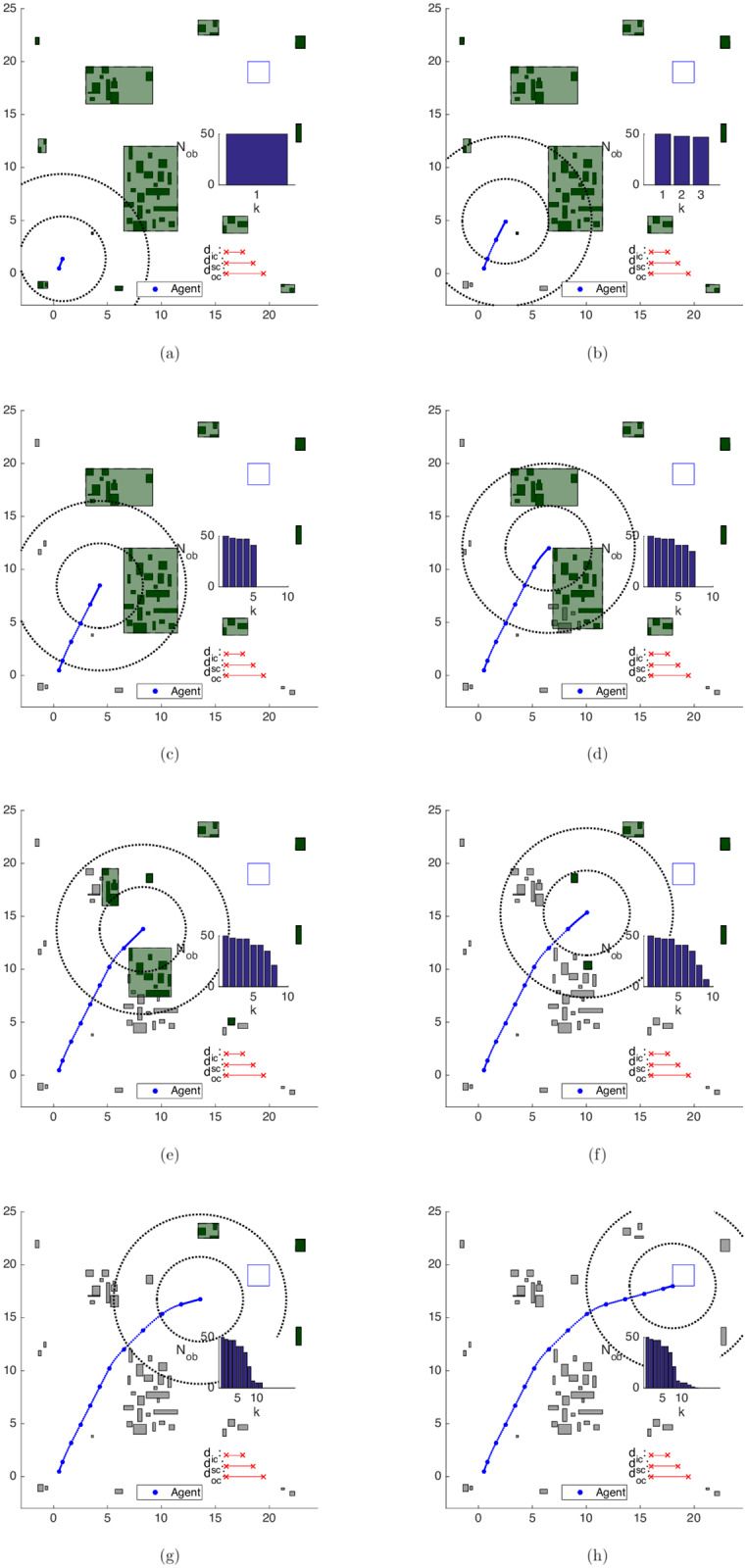
Trajectory of the bygone obstacles clustering strategy in the map of Fig 17a. (a) Step 1. (b) Step 3. (c) Step 5. (d) Step 7. (e) Step 8. (f) Step 9. (g) Step 11. (h) Step 14.

In Fig 22 we have the clustering strategy with the *rebuttal of bygone obstacles* as the agent moves towards the goal area, *i.e*. running Alg 7.

**Fig 23:**
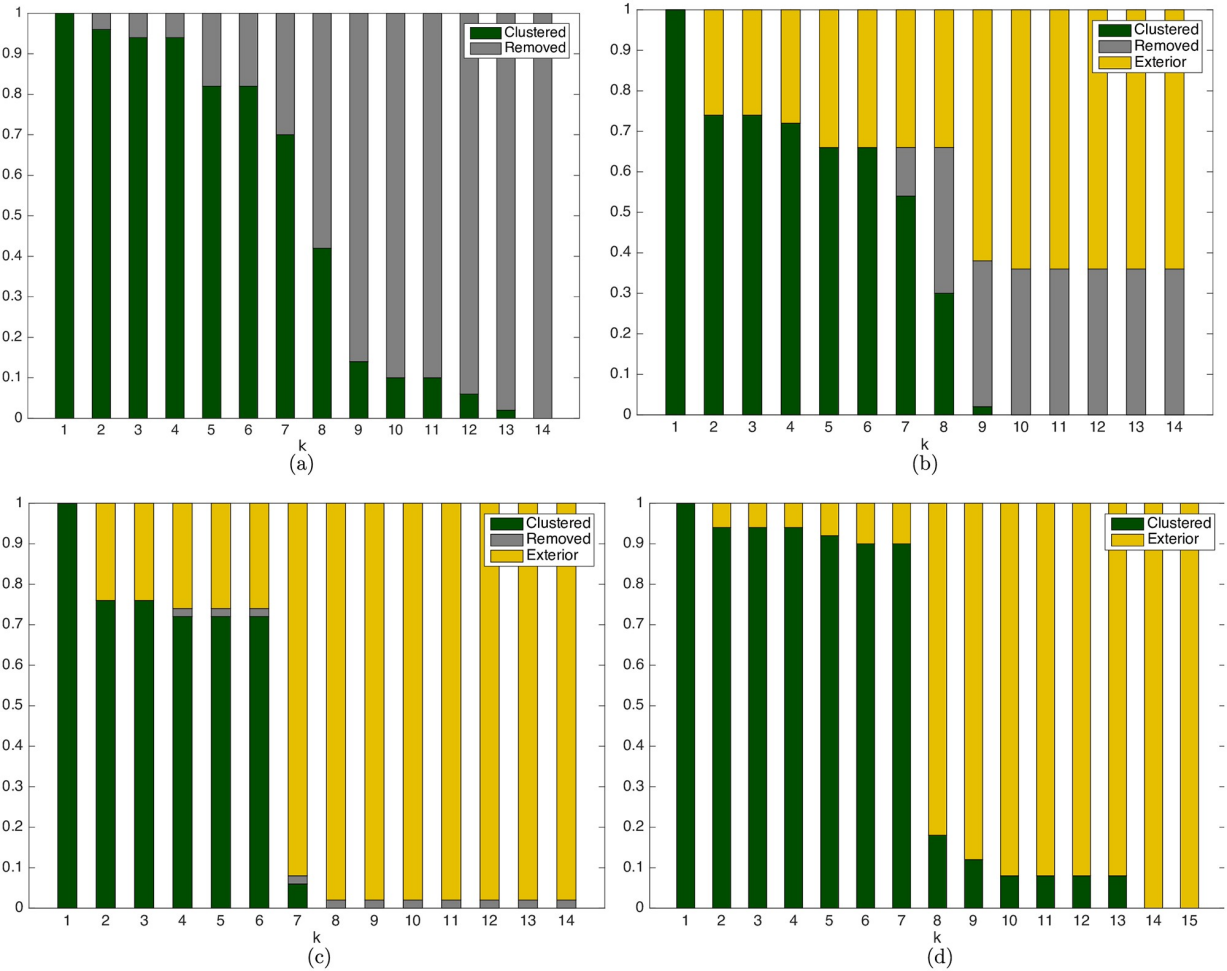
Obstacle classification for the bygone, exterior and iterative obstacle clustering strategies. (a) Bygone Obstacles Rebuttal strategy. (b) Exterior Obstacles Contempt strategy. (c) Iterative Clustering Distance Tuning strategy (d) Iterative Clustering Distance Tuning strategy in the random obstacles scenario.

In the beginning, the trajectory of Fig 22a is the same as the one of Fig 20a, once this strategy only removes obstacles that are already distancing from the agent and will do so thenceforth. This approach gradually replaces these obstacles by simple convex constraints once the agent is predicted to always move away from them, as the drop in *N*_*ob*_ to the right-hand side of Fig 22b–22h shows, with respectively $N_{ob}^{p}=3,9,15,29,$ 43, 45 and 50 bygone obstacles, represented in *grey* in each figure. The evolution of the classification of *N*_*ob*_ can be seen in Fig 23a.

The decrease of the computational load taken as input of the clustering procedure can be seen both in the representation of *N*_*ob*_, at the right-hand side of every plot along Fig 22a–22h, as in the obstacle classification in Fig 23a for all the simulation steps.

The average time spent on each algorithm iteration is shown in Fig 21b. Along *all* steps of the trajectory, the average time necessary to identify the *bygone* obstacles is $t_{B,b}\approx 0.01\;s$ and the average clustering time is $t_{K,b}\approx 0.08\;s$. With the Bygone obstacles rebuttal strategy, the average total simulation time, *i.e*. the average of the summation of *all* the optimization times until the target set was reached is *t*_*b*_ ≈ 3.14 *s*, with an average nominal speedup of *S*_*b*_ ≈ 19.64 with regard to the Unclustered strategy.

### 6.4 Exterior obstacles contempt strategy

**Fig 24:**
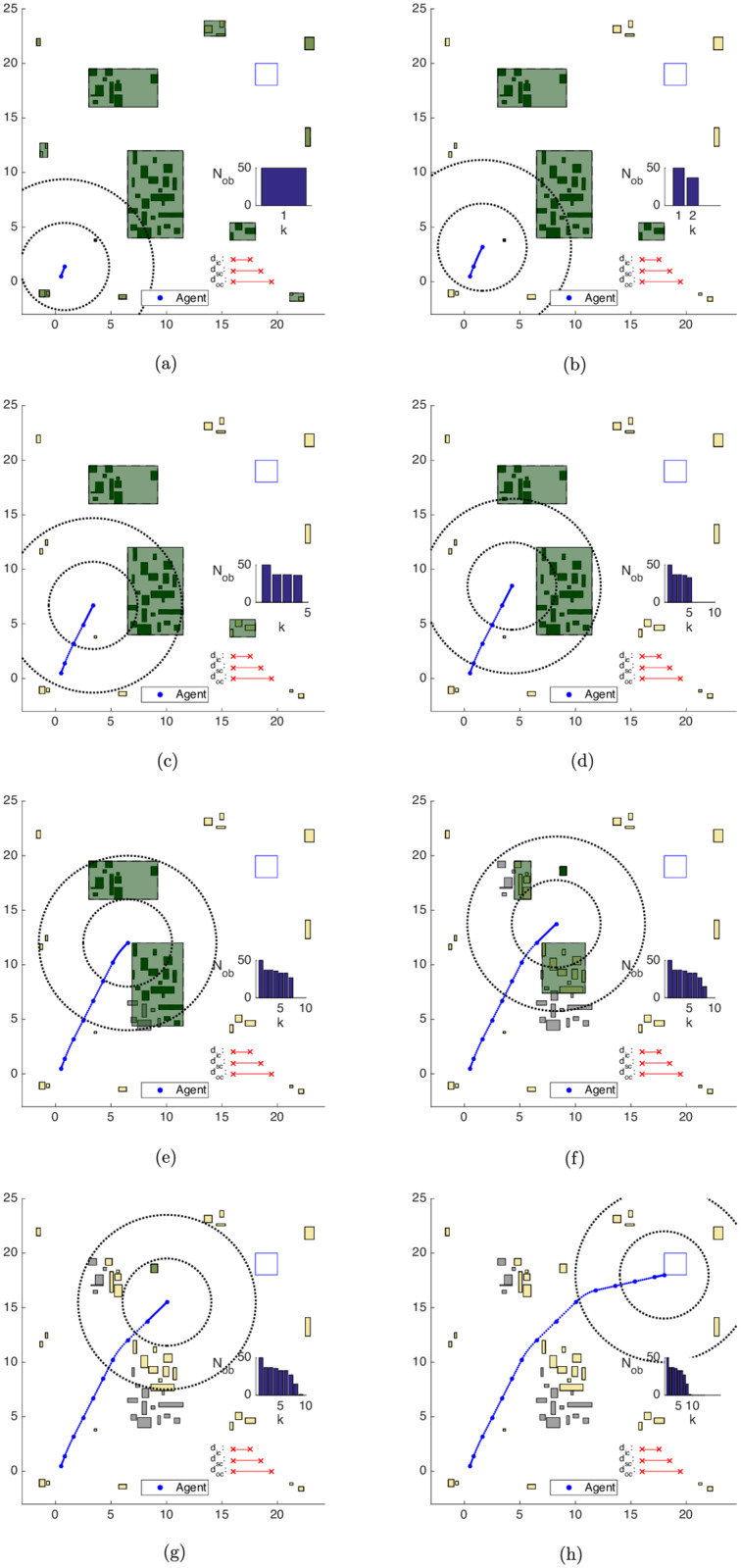
Trajectory of the exterior obstacles contempt strategy in the map of Fig 17a. (a) Step 1. (b) Step 2. (c) Step 4. (d) Step 5. (e) Step 7. (f) Step 8. (g) Step 9. (h) Step 14.

In Fig 24a, the Exterior obstacles clustering strategy (Alg 10) initially identifies $N_{ob}^{e}=13$ exterior obstacles. We represent them in green-yellowish color in the timestep the algorithm identifies them and in yellow color in the subsequent steps.

One can see that the *exterior* obstacle contempt strategy complements the effects of the *bygone* obstacle rebuttal one. The obstacles that belong to *exterior* clusters are promptly removed after the first step, and while the agent circumvents both the central and northwestern clusters, some obstacles of these clusters become *bygone* obstacles before they are treated as *exterior* ones. See the obstacles in the lower-half of the central cluster and to the left-hand side of the north-west cluster in Fig 24f–24g. As the agent surpasses both clusters, the predicted (future) trajectory stands at the same side of them and turns every remaining obstacle in both clusters into exterior ones. This causes the drop of *N*_*ob*_ observed in *k* = 9 from Fig 24g on. The maneuver ends with $N_{ob}^{e}=32$ exterior obstacles and $N_{ob}^{p}=18$ *bygone* obstacles in Fig 24h, and the evolution of the classification of *N*_*ob*_ can be seen in Fig 23b.

Along *all* steps of the trajectory, the average time required to identify the exterior obstacles is $t_{E,e}\approx 0.00\;s$, the average identification time of bygone obstacles is $t_{B,e}\approx 0.00\;s$ and the average clustering time is $t_{K,e}\approx 0.05\;s$. With the exterior obstacles clustering strategy, the average total simulation time, *i.e*. the average of the summation of *all* the optimization times until the target set was reached is *t*_*e*_ ≈ 1.61 *s*, with an average nominal speedup of *S*_*e*_ ≈ 38.33 with respect to the Unclustered strategy.

### 6.5 Iterative clustering distance tuning

**Fig 25:**
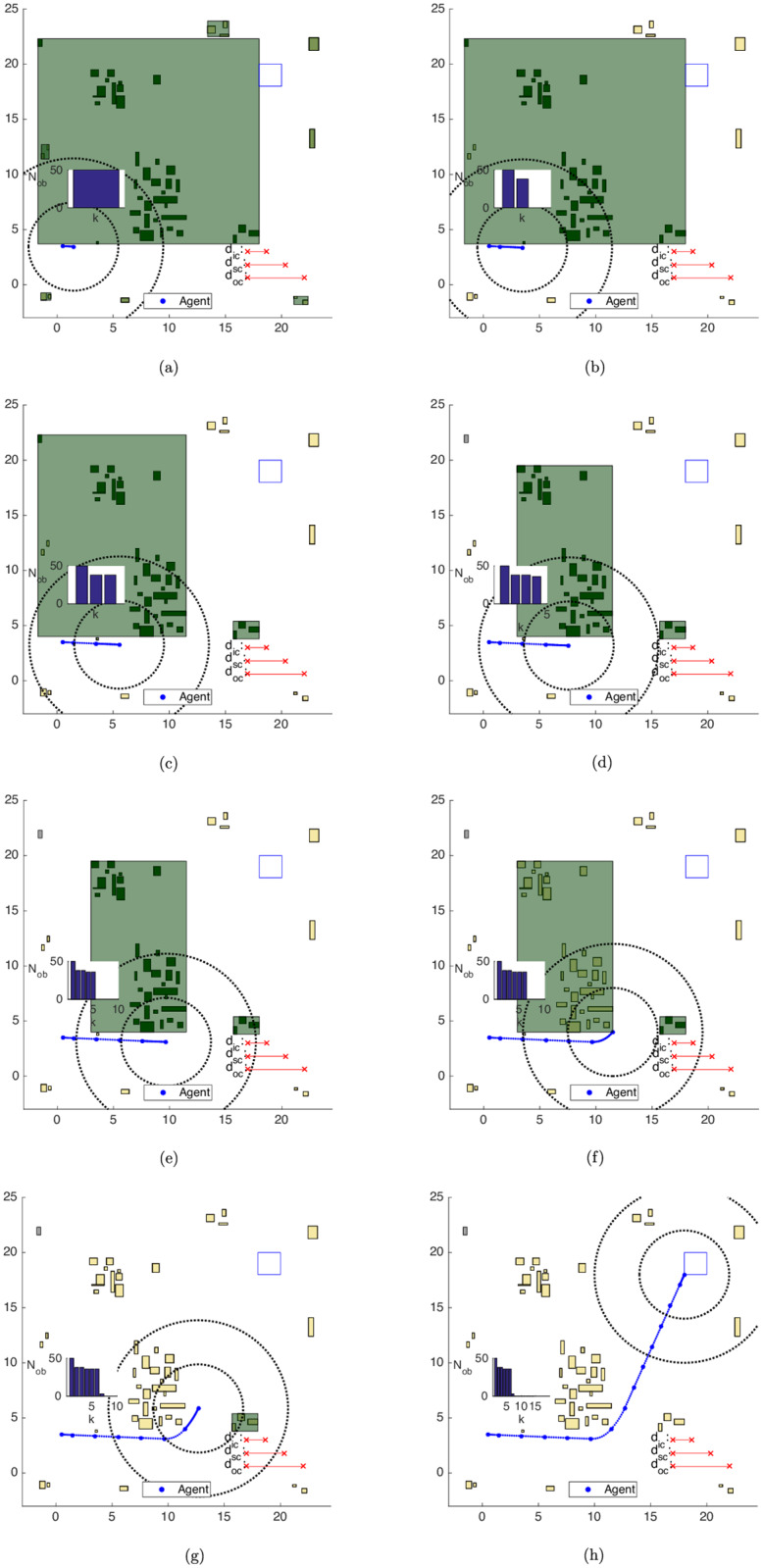
Trajectory of the iterative clustering distance tuning strategy in the map of Fig 17a. (a) Step 1. (b) Step 2. (c) Step 3. (d) Step 4. (e) Step 5. (f) Step 6. (g) Step 7. (h) Step 14.

The results for the Iterative Clustering Distance Tuning Strategy in Alg 11 can be seen in Fig 25. The agent initial state is set as **x**_**0**_ = [0.5 0 3.5 0]^*T*^ (*i.e*. the agent is brought closer to the obstacle set) and the first guess for the clustering distance is increased to $d_{c_{0}}={\left[3\;6\;9\right]}^{T}$, to ensure the agent is initially surrounded by a cluster. The shrinking rate is taken as *s*_*r*_ = 0.75.

Initially, the algorithm groups the obstacles into a cluster configuration that covers the entire territory, which is not shown in Fig 25. Since no movement is possible, as a single cluster surrounds the agent and prevents it from choosing a viable action, the algorithm shrinks the clustering parameters. However, a second infeasible problem arises, which demands another reclustering procedure.

The result is the maneuver of Fig 25a, with the agent moving south to leave the avoidance area of the largest cluster. As the agent begins to circumvent it, after Fig 25b, its central-south obstacles enter into the *surrounings-zone*. The agent makes a less sharp curve with the new open area between the clusters and the trajectory ends with $N_{ob}^{p}=1$ and $N_{ob}^{e}=49$.

The average times needed along the first and the second reclustering steps, which *comprise* the time CPLEX needed to conclude that the problem is infeasible, are $t_{R,i,1}\approx t_{R,i,2}\approx 0.01\;s$. Along *all* steps of the trajectory, the average time required to identify exterior obstacles is $t_{E,i}\approx 0.00\;s$, the average identification time of bygone obstacles is $t_{B,i}\approx 0.00\;s$ and the average clustering time is $t_{K,i}\approx 0.05\;s$.

With the Iterative clustering distance tuning strategy, the average total simulation time, *i.e*. the average of the summation of *all* optimization times until the target set was reached is *t*_*i*_ ≈ 1.03 *s*, with an average speedup of *S*_*i*_ ≈ 59.53 with regard to the results of the Unclustered strategy.

### 6.6 Iterative clustering distance tuning in the random obstacle scenario

**Fig 26:**
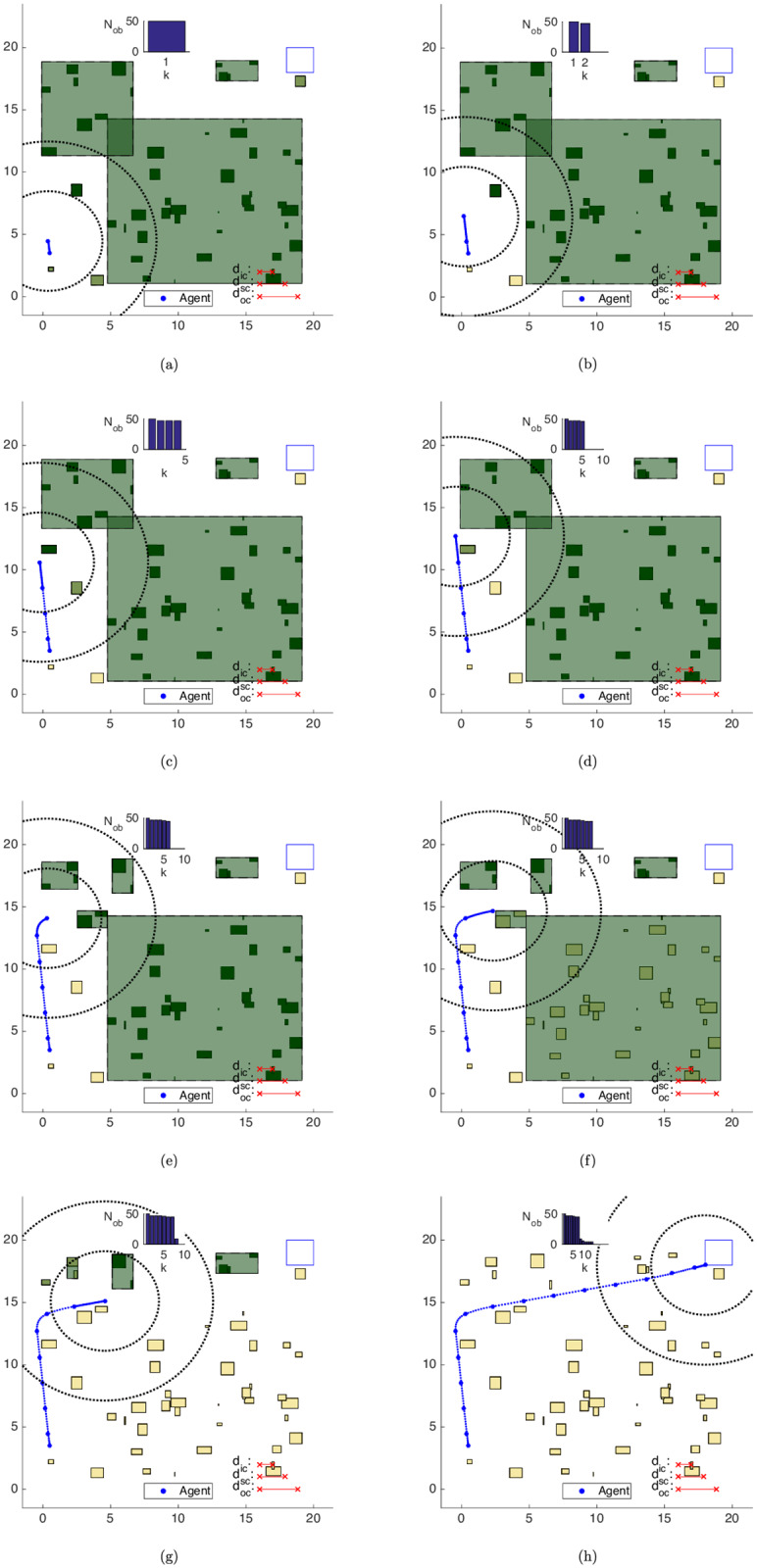
Trajectory of the iterative clustering distance tuning strategy in the map of Fig 17b. (a) Step 1. (b) Step 2. (c) Step 4. (d) Step 5. (e) Step 6. (f) Step 7. (g) Step 8. (h) Step 15.

We used the Close, Bygone, Exterior and the Iterative Clustering strategies in another environment, with obstacles randomly generated according to Eq (22) for *N*_*s*_ = 18 *steps*, [*f*_0_, *f*_1_] = [0, 20] and *m* = 0. Now there are 50 small uniformly-distributed obstacles in the environment and Fig 26 contains the results. The shrinking rate is kept as *s*_*r*_ = 0.75.

The algorithm adjusts the initial clustering distance 4 times, what takes respectively $t_{R,r,1}\approx t_{R,r,2}\approx t_{R,r,3}\approx 0.00\;s$ and $t_{R,r,4}\approx 0.01\;s$ in each reclustering operation, which *includes* the time CPLEX needed to deduce that the problem is infeasible, until the 50 obstacles are arranged in Fig 26a into 7 clusters for *d*_*c*_ ≈ [0.95 1.90 2.85]^*T*^.

**Fig 27:**
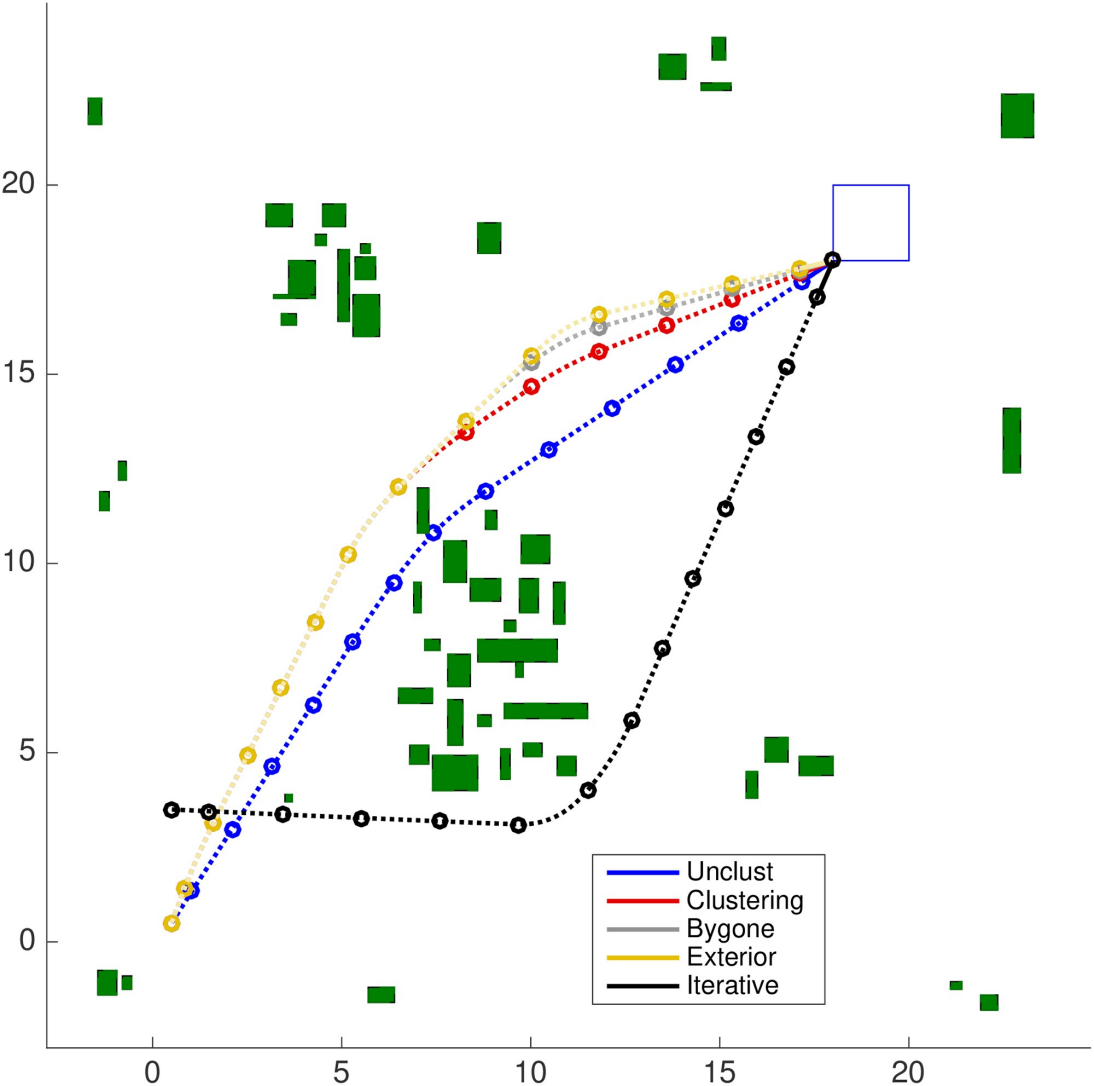
Trajectory comparison in the map of Fig 17a for the unclustered, clustering, bygone, exterior and iterative strategies. The proximity in respect to the euclidean distance with the Unclustered path is given in decreasing order by the Clustering, Bygone, Exterior and Iterative strategies.

With the Iterative clustering distance tuning strategy, the average total simulation time, *i.e*. the average of the summation of *all* simulation times until the agent reaches the target set is *t*_*r*_ ≈ 2.63 *s*, with an average speedup of *S*_*r*_ ≈ 13.43 in relation to the results of the Unclustered strategy in the random scenario. A comparison of the trajectory that these strategies obtained can be seen in Fig 27 and is discussed in detail in the Discussion section.

## 7 Discussion

### 7.1 Obstacle classification

The obstacle composition for the cases studied in this work can be seen in Fig 23. The main tendency we can observe is that the more elaborate the strategy, the faster the substitution of binary variables of obstacle avoidance. While the Bygone obstacle clustering strategy in Fig 23a replaces all binary avoidance constraints in *k* = 14, the addition of the Exterior clustering strategy achieves the same in *k* = 10 and the adoption of the Iterative tuning heuristics accomplishes it in *k* = 7. These strategies cooperate in the substitution of binary avoidance variables, and the preponderance of any of them over the others is intrinsically related both to the clustering distances adopted and the obstacle topology in the environment, as well as the order in which they are carried out. However, one practical limitation of these algorithms is the need for full knowledge of the obstacle environment topology upon which they will operate.

### 7.2 Evolution of the cost function and its prediction

**Fig 28:**
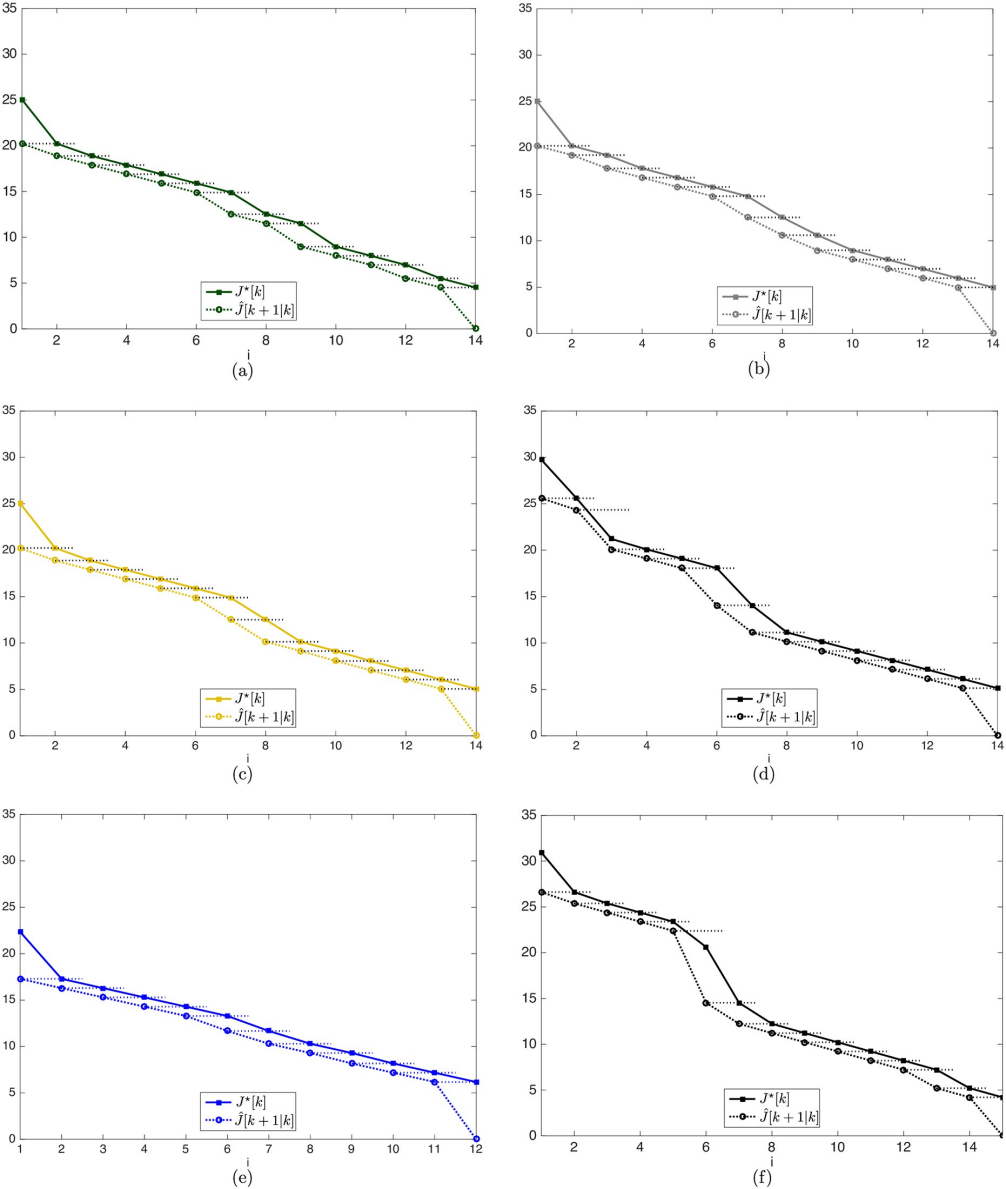
Evolution of the cost function and its prediction for the clustering, bygone, exterior and iterative strategies. (a): Clustering strategy in the map of Fig 17a. (b): Bygone obstacles clustering strategy in the map of Fig 17a. (c): Exterior obstacles clustering strategy in the map of Fig 17a. (d): Iterative clustering strategy in the map of Fig 17a. (e): Unclustered strategy in the map of Fig 17b. (f): Iterative clustering strategy in the map of Fig 17b.

In Fig 28 there is a representation of the cost function evolution, in continuous lines, and its prediction, in dashed lines, along all the simulations presented here.

Along all the steps of the Close, Bygone, Exterior and Unclustered strategies, the cost function *J*^⋆^[*k*] corresponds exactly to the prediction $\hat{J}\left[k+1|k\right]$ made in the *previous* step, as the dashed horizontal extensions in the value of $\hat{J}\left[k+1|k\right]$ show. However, in the Iterative strategy, both for *k* = 3 in Fig 28d and for *k* = 6 in Fig 28f, when an obstacle occupies an inner clustering region, the clustering algorithm frees previously clustered space that becomes available for the agent movement, which makes the value of the cost function be less than the prediction previously made.

Such fact was observed here only in the Iterative clustering strategy, but it is worth noting that it is a consequence of the distribution of the *d*_*c*_ values in the clustering regions around the agent. With a fine parameter tuning, the Close strategy itself would identify the same phenomenon.

### 7.3 Trajectory comparison

In Fig 27 we can compare the trajectories that each strategy obtains in the map of Fig 17a. While the Unclustered strategy optimizes a trajectory that overcomes the obstacles that compose the central cluster through an internal path, the beginning of the Clustering, Clustering + Bygone and Clustering + Bygone + Exterior strategies is exactly the same, moving north to circumvent it.

After surpassing the central cluster at time *t* = *k*_0_+ 8, the trajectories slightly separate themselves until they rejoin at the lower left corner of Q, in *t* = *k*_0_+ 14. In this case, the *Exterior* strategy takes a path that prioritizes the vertical displacement to the detriment of the horizontal one, whereas the *Bygone* and the *Clustering* strategies take paths more similar to a straight line toward the target.

The *Iterative* clustering strategy in *black*, on the other hand, chooses the first available path freed in the iterative deepening search among the obstacles. Thereby, the trajectory of the agent overcomes the central cluster underneath.

In Fig 27, the comparison of the paths the Clustering, Bygone, Exterior and Iterative strategies obtained against the one optimized by the Unclustered strategy alone shows that the more comprehensive the changes in the original planning problem, the more the final trajectory potentially diverges from the optimal path. In such case, there is an increment of approximately 2.8%, 2.8%, 2.8% and 21.2% in the the value of the cost function with the adoption of these strategies.

**Fig 29:**
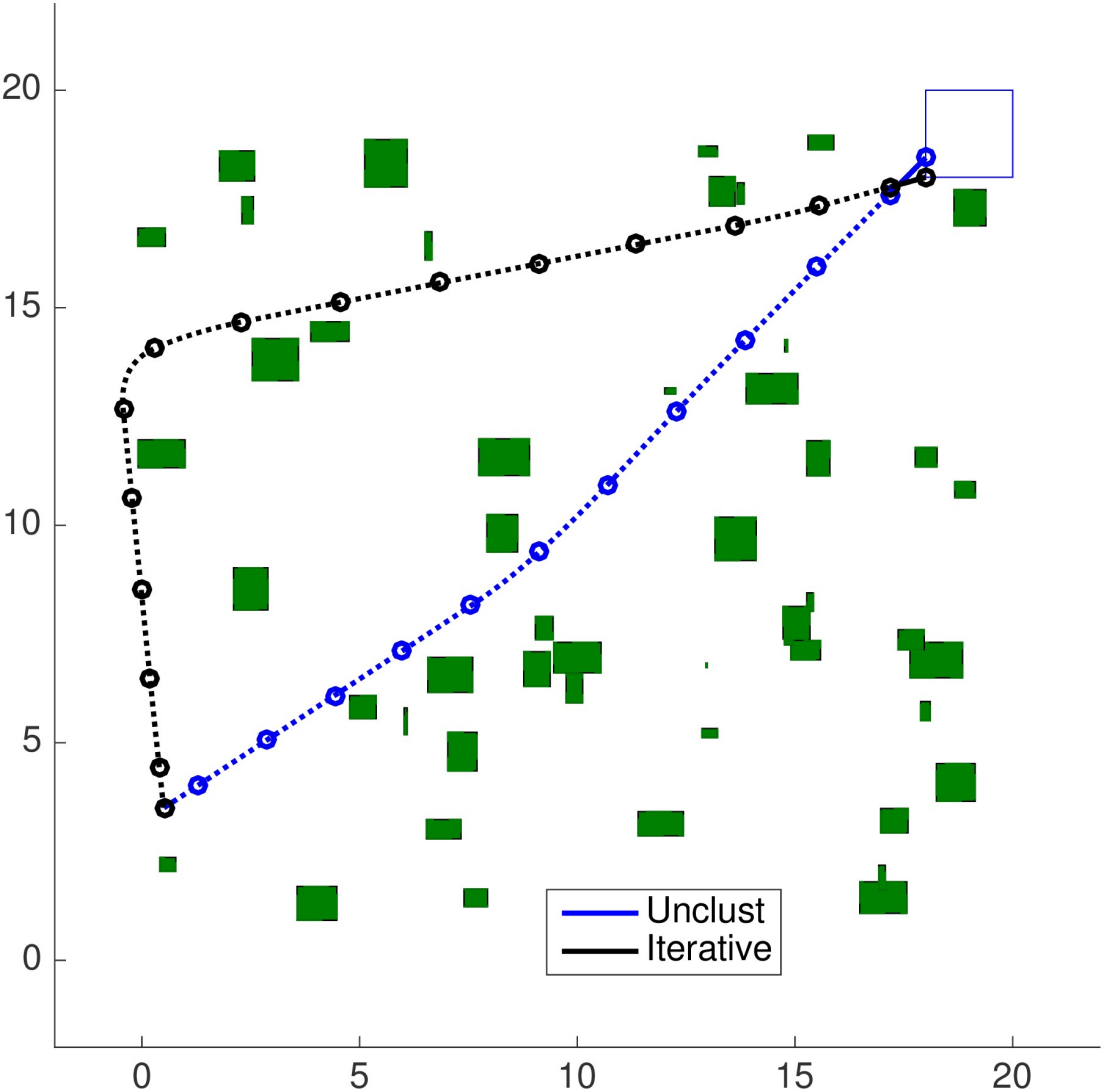
Trajectory comparison in the map of Fig 17b for the unclustered and iterative strategies. While the optimal path found by the Unclustered strategy approaches a straight-line among the obstacles, the path that the Iterative strategy obtains initially moves northwards to escape the northwestern cluster.

In Fig 29 we have a comparison of the trajectories that the Iterative and the Unclustered strategies optimize in the random scenario of Fig 17b. The Iterative strategy obtains the nozzle-shaped trajectory shown in *black*, which shows that the path produced can substantially differ from the optimal one, as a consequence of the different constraints that make up the problem. Here, there is an increment of approximately 30.7% in the value of the cost function with the adoption of the clustering strategies.

We will see in the next subsection what are the outcomes of the adjustments made in the original planning problem, specially regarding the optimization times.

### 7.4 Computational time

The strategies proposed in this paper have two main effects in the agent trajectory. The first one is their impact on the resulting trajectories, as discussed in the last subsection. The second consequence is the decrease in the optimization times, the main drawback in a Mixed Integer problem and the primary advantage of our approach.

We summarize in Table 2 the resulting computational times of the presented strategies on the map of Fig 17a. For every strategy we represent both the nominal time of the CPLEX optimization—in the second column—as well as the Total time necessary to obtain the complete trajectory—in the seventh one. The latter includes *all* operations performed during the solution of the computational problem with the only exception of loading it initially into main memory. Additionally, we also identify the *aggregated* time, *i.e*. the time of the operations performed along all time steps from the initial position until the agent reaches the target set, that each strategy spends to obtain not only the Clustering configuration, but also the Bygone and Exterior constraints and the *overall* time spent on Infeasible iterations.

**Table 2:** Average computational times and speedup for the map of Fig 17a.

Fig 17a	Average time [s]						Speedup	
Strategies:	CPLEX	Clustering	Byg. Constr.	Ext. Constr.	Infeasib.	Total	Nominal	Effective
**Unclustered**	61.653	–	–	–	–	2708.2	1	1
**Clustering**	3.792	0.179	–	–	–	51.835	16.260	52.246
**Bygone**	3.139	0.077	0.007	–	–	18.189	19.643	148.892
**Exterior**	1.609	0.048	0.002	0.002	–	6.066	38.327	446.441
**Iterative**	1.036	0.046	0.000	0.002	0.012	2.718	59.527	996.321
**Relaxed**	0.736	0.043	0.005	0.000	–	1.160	–	–

Average times spent in the CPLEX optimization and the *aggregated* time to obtain the Clustering configuration, the Bygone Constraints, the Exterior Constraints, the *overall* time spent of Infeasible iterations and the Total simulation time.

The trajectory of the Relaxed strategy is a straight-line from the initial agent position to the target set. In this case, the agent behaves as if there is *no* obstacle to be avoided. Naturally, it is not possible to attain a faster approach. Despite not being drawn in Figs 27 and 29, its solution time can be used as a benchmark to obtain the *lower-bound* on the optimization times.

From the data in Table 2, the main inference is that the extra time spent in the techniques we propose is negligible when compared to the total simulation time. The CPLEX optimization time falls progressively as more elaborate clustering strategies are used because the effect of each new component adds up incrementally to the previous ones.

The *Effective* speedup column measures the typical behavior of each strategy in *practical* situations. It is no use to guarantee speedup only in the problem solving phase, the so-called *Nominal* speedup here, if we add overhead in other steps, transferring computational load to stages not monitored and *previous* to the main computation. It is necessary to decrease the *total* solution time, and from Table 2 we can see that the *Nominal* speedup propagates to other solving phases, which renders an *Effective* reduction in the computing times of approximately 52, 148, 446 and 996 times for the *Close*, Bygone, *Exterior* and *Iterative* strategies, respectively.

All the strategies presented here offer both substantial *Nominal* and *Effective* speedups regarding the Unclustered scenario. Besides that, the *Exterior* and *Iterative* strategies also offer simulation times in the *same order* of magnitude than the *lower-bound* offered by the Relaxed strategy, which shows the effectiveness of the techniques proposed.

The same results for the Unclustered and Iterative Strategies in the Random environment of Fig 17b are shown in Table 3. Finally, the times spent on reclustering steps in the Iterative strategy are represented in Table 4. represented in Table 4.

**Table 3:** Average computational times and speedup for the map of Fig 17b.

Fig 17b	Average time [s]						Speedup	
Strategies:	CPLEX	Clustering	Byg. Constr.	Ext. Constr.	Infeasib.	Total	Nominal	Effective
**Unclustered**	35.274	–	–	–	–	2676.8	1	1
**Iterative**	2.627	0.074	0.000	0.002	0.013	5.796	13.429	461.804
**Relaxed**	0.713	0.051	0.006	0.000	–	1.051	–	–

Average times spent in the CPLEX optimization and the *aggregated* time to obtain the Clustering configuration, the Bygone Constraints, the Exterior Constraints, the *overall* time spent of Infeasible iterations and the Total simulation time.

**Table 4:** Average time spent on reclustering iterations.

Map:	Iteration 1 [s]	Iteration 2 [s]	Iteration 3 [s]	Iteration 4 [s]
Fig 17a	0.005	0.007	–	–
Fig 17b	0.002	0.004	0.002	0.005

Average time spent on reclustering iterations.

## 8 Conclusion

In this work we proposed a combination of approaches that guaranteed performance improvement through the reduction of the computational load in large scale trajectory planning methods with obstacle avoidance techniques. While traditional methods find the globally optimal path via a complete search in the solution space, the technique we proposed pruned areas that belonged to the constrained domain, which eased the exponential burden related to obstacle avoidance. This improvement dealt directly with the computation of the *complete* path of an agent, *i.e*. without the definition of any waypoint, which could have resulted in faster computational times. Through a deferred decision-based technique, we tackled the computationally heavier problems only if necessary, reducing the solution search time.

We proposed an algorithm that takes into account the pertinence of each obstacle, based on its temporal relevance to the agent guidance problem. This strategy offered computational speedup based solely on problem modeling, *i.e*. independent of the computer architecture or the variable encoding chosen, which naturally opened space for further improvements on subsequent research.

With the clustering of obstacles, the number of regions to be avoided can be reduced offline and has the theoretical lower limit of one. The reduction in the number of regions to be avoided entails a lower computational load online. Since the clustering algorithm is cheap and runs offline, this yields overall better computational times. However, the clusters impact performance in terms of the cost function, as the optimal solution without clustering might not be feasible anymore. Therefore, one identifies a compromise between optimality and computational burden. The iterative clustering distance tuning enables to automatically find clustering distances that are quasi-minimal to obtain feasibility of the optimization problem with clustering.

The bygone obstacles rebuttal and the exterior obstacles contempt strategies require a previous solution to the optimization problem, therefore they cannot be run offline before the optimization. On the other hand, these two strategies remove obstacles that do not affect the optimal trajectory, therefore optimality is maintained.

As future research proposals we can relate: extension to *multiple* agents; optimization of the cluster configuration; development of an obstacle avoidance strategy with anytime algorithm capacity; use of other techniques, such as complex networks to identify the clusters or metaheuristics to generate convex regions to speedup the MIP solving phase; use of different encodings to improve the solver performance with regard to the obstacle avoidance; and the study of the effect of uncertainty in the model through the use of robust optimization and fuzzy programming.

## Notation

$X\subset R^{4}$: polytope of admissible state values
$U\subset R^{2}$: polytope of admissible control values that contains the origin
x∈X: plant state
$x_{0}\in X$: initial plant state
u∈U: control input
$r\in R^{2}$: agent position
k∈N: current time
$\hat{⊙}\left[k+\alpha |k\right]$: predicted value of ⊙ in time *k*+ *α* based on information available up to *k*
N[k]∈N: MPC control and prediction horizon
$Q\subset R^{4}$: polytope of terminal state values
γ⊂R: weight of the term associated with fuel consumption in the cost function
$N_{Q}\in N$: number of sides of the target set
$M_{t}\in R_{+}$: constant large enough to make terminal constraints inactive
$N_{f}\in N$: number of faces of the obstacles
$O\subset R^{4}$: polytopic obstacle
$\bar{N}\in N$: maximal horizon
*b* ∈ {0, 1}: binary variable associated to horizon minimization
$M_{o}\in R_{+}$: constant large enough to make obstacle and intersample avoidance constraints inactive
$N_{ob}\in N$: number of obstacles in T
$R_{ob}\in R^{N_{f}N_{ob}}$: coordinates of the obstacles
$T\subset R^{2}$: territory that contains obstacles
$b_{f,k}^{O}\in \left\{0,1\right\}$: binary variables associated to the obstacle avoidance constraints
$N_{c}\in N$: number of clusters in T
$R_{\kappa }\in R^{N_{f}N_{c}}$: coordinates of the clusters
$\Omega \in R^{2}$: edges that connect neighboring obstacles
$O\in R^{N_{f}}$: obstacle set with coordinates *R*_*ob*_
G=(O,Ω): undirected graph of the obstacle set O with $\left|O\right|=N_{ob}$ vertices and |Ω| edges
$d_{c}^{max}\in R$: maximum clustering distance for some given clustering region
$r_{d}\left(p_{\cdot }\right)\in R^{2}$: position values of point *p*_⋅_
$A\in {\left\{0,1\right\}}^{N_{ob}\times N_{ob}}$: adjacency matrix of a graph with *N*_*ob*_ obstacles
$N_{d}\in N$: number of nodes in the graph
$C\in {\left\{0,1\right\}}^{N_{ob}\times N_{ob}}$: connectivity matrix of a graph with *N*_*ob*_ obstacles
$d\in R_{+}$: Hausdorff distance between obstacles
$d_{c}\left[\mu \right]\in R^{N_{ob}}$: interobstacle clustering distance of the *μ*-th obstacle
$R_{ic}\in R$: radius of the inner zone clustering region
$R_{sc}\in R$: radius of the surroundings zone clustering region
$d_{ic}\in R$: clustering distance of the inner zone clustering region
$d_{sc}\in R$: clustering distance of the surroundings zone clustering region
$d_{oc}\in R$: clustering distance of the outer zone clustering region
$\Delta r_{ao}\in R^{N_{ob}}$: agent-obstacle distances
$\Delta r_{o}\in R^{N_{ob}\times N_{ob}}$: interobstacle distances
$\tilde{C}\in {\left\{0,1\right\}}^{N_{c}\times N_{ob}}$: connected components matrix of a graph with *N*_*ob*_ obstacles and *N*_*c*_ clusters
$R_{f}\in R^{4}$: coordinates of the projection of Q onto the position space
$P_{Q}$: projection of Q onto the position space
$N_{s}\in N$: number of steps used in the simulation
$r_{x}\in R$: position along a coordinate axis in a horizontal plane regarding an arbitrary origin
$r_{y}\in R$: position along a coordinate axis (perpendicular to the first) in a horizontal plane regarding an arbitrary origin
$v_{x}\in R$: velocity regarding the *r*_*x*_ position
$v_{y}\in R$: velocity regarding the *r*_*y*_ position
$a_{x}\in R$: acceleration regarding the *v*_*x*_ velocity
$a_{y}\in R$: acceleration regarding the *v*_*y*_ velocity
$T\in R_{+}$: sample period in time units
$L_{z}\in R$: dimension of the random obstacle environment in the same axis as the one of *r*_*z*_, where *r*_*z*_ refers to either the same axis of *r*_*x*_ or *r*_*y*_
$b_{z}\in R$: relative coordinate of the obstacle center in the same axis as the one of *r*_*z*_, where *r*_*z*_ refers to either the same axis of *r*_*x*_ or *r*_*y*_
$h_{z}\in R$: average length of a random obstacle in the same axis as the one of *r*_*z*_, where *r*_*z*_ refers to either the same axis of *r*_*x*_ or *r*_*y*_
$B_{z}\in R$: coordinate of the center of the obstacle regarding an arbitrary origin, where *z* refers to either the same axis of *r*_*x*_ or *r*_*y*_
$f_{0z}\in R$: position of the smallest coordinate of the environment regarding an arbitrary origin, where *z* refers to either the same axis of *r*_*x*_ or *r*_*y*_
$f_{1z}\in R$: position of the largest coordinate of the environment along the same coordinate axis of *f*_0*z*_
$m_{z}\in R$: length of a border which defines the region that contains the obstacle set, where *z* refers to either the same axis of *r*_*x*_ or *r*_*y*_
$N_{ob}^{b}\in N$: number of *bygone* obstacles in a territory
$N_{ob}^{e}\in N$: number of exterior obstacles in a territory
